# Nanoparticles containing constrained phospholipids deliver mRNA to liver immune cells in vivo without targeting ligands

**DOI:** 10.1002/btm2.10161

**Published:** 2020-05-27

**Authors:** Zubao Gan, Melissa P. Lokugamage, Marine Z. C. Hatit, David Loughrey, Kalina Paunovska, Manaka Sato, Ana Cristian, James E. Dahlman

**Affiliations:** ^1^ Wallace H. Coulter Department of Biomedical Engineering Georgia Institute of Technology and Emory School of Medicine Atlanta Georgia USA

**Keywords:** drug delivery, dna barcoding, gene therapies, LNP, mRNA

## Abstract

Once inside the cytoplasm of a cell, mRNA can be used to treat disease by upregulating the expression of any gene. Lipid nanoparticles (LNPs) can deliver mRNA to hepatocytes in humans, yet systemic non‐hepatocyte delivery at clinical doses remains difficult. We noted that LNPs have historically been formulated with phospholipids containing unconstrained alkyl tails. Based on evidence that constrained adamantyl groups have unique properties that can improve small molecule drug delivery, we hypothesized that a phospholipid containing an adamantyl group would facilitate mRNA delivery in vivo. We quantified how 109 LNPs containing “constrained phospholipids” delivered mRNA to 16 cell types in mice, then using a DNA barcoding‐based analytical pipeline, related phospholipid structure to in vivo delivery. By analyzing delivery mediated by constrained phospholipids, we identified a novel LNP that delivers mRNA to immune cells at 0.5 mg/kg. Unlike many previous LNPs, these (a) did not preferentially target hepatocytes and (b) delivered mRNA to immune cells without targeting ligands. These data suggest constrained phospholipids may be useful LNP components.

## 1 INTRODUCTION

Adamantane, a cage saturated hydrocarbon consisting of three linked cyclohexane rings, forms a rigid diamond‐like structure. As a result, adamantane derivatives can exhibit high thermal stability,^1^ wide optical transparency, and superior mechanical and electrical properties.^2^ These properties have led to the application of adamantane derivatives in materials science and nanotechnology. Adamantanes are also used in small molecule drugs, most notably antivirals including amantadine, rimantadine and tromantadine,^3^ due to two traits. First, their structure can alter the interactions between the small molecule and cell‐surface proteins. For example, Amantadine, an aminoadamantane, was found to disrupt the fusion between the influenza virus and the host cell by inhibiting the M2 ion channel protein, which is vital for viral access.^4^ Second, due to their lipophilicity, adamantyl groups can improve drug pharmacokinetics.^5^ Specifically, the conformationally constrained structure of the adamantyl group protects nearby functional groups from metabolic cleavage, thereby improving drug stability.^6^ These properties have made adamantyl groups useful for medicinal chemists targeting influenza,^7^, ^8^ HIV^9^, ^10^ hepatitis C virus,^11^ and Parkinson's disease.^12^

Both traits described above (protein interactions and lipophilicity) impact the efficacy and safety of lipid nanoparticles (LNPs), which have shown promise in delivering RNA^13^, ^14^ to hepatocytes in humans. One key limitation in the field of RNA therapies is systemic non‐hepatocyte RNA delivery, which has remained challenging. We recently reported that LNPs with adamantyl‐containing ionizable lipids, referred to as “constrained LNPs”, delivered siRNA to splenic T cells without an active targeting ligand.^15^ These data demonstrated that incorporating adamantyl groups into ionizable lipids can lead to interesting LNP tropism in vivo. However, we were unable to identify whether (a) LNPs containing adamantyl groups delivered mRNA in vivo and (b) adamantyl groups could function when incorporated into other LNP components. Specifically, LNPs are comprised of up to four components: ionizable lipids, PEG‐lipids, cholesterols, and phospholipids. Several reports have shown that phospholipid structure can affect the tropism and efficacy of LNPs in vivo.^16^, ^17^ Based on this report, we investigated the structure of phospholipids used in previous LNPs. We noted that phospholipids in previously reported LNPs have not utilized constrained lipid tails. Given the chemical properties of adamantane described above, we hypothesized that adamantyl phospholipids could be incorporated into LNPs that delivered mRNA in vivo.

## 2 RESULTS

To test this hypothesis, we synthesized a series of novel adamantyl‐phospholipids and quantified how 109 LNPs functionally delivered mRNA (i.e., mRNA translated into protein) to 16 cell‐types in mice (i.e., in vivo). This systematic high throughput in vivo assay^18^, ^19^, ^20^ forgoes traditional cell culture experiments. Our data suggest performing drug delivery studies directly in vivo is important; specifically, we found that in vitro nanoparticle delivery can be a poor predictor of in vivo nanoparticle delivery.^21^ The DNA barcoding‐based system enables us to quantify how >100 nanoparticles functionally deliver mRNA (i.e., mRNA translated into protein) in any combination of cell types, in a single mouse. Subsequently analyzing the relationship between adamantyl‐phospholipid structure and in vivo delivery using a DNA barcoding‐based analytical pipeline, we identified a novel LNP containing constrained phospholipids that delivers mRNA to immune cells in vivo without targeting ligands.

**FIGURE 1:**
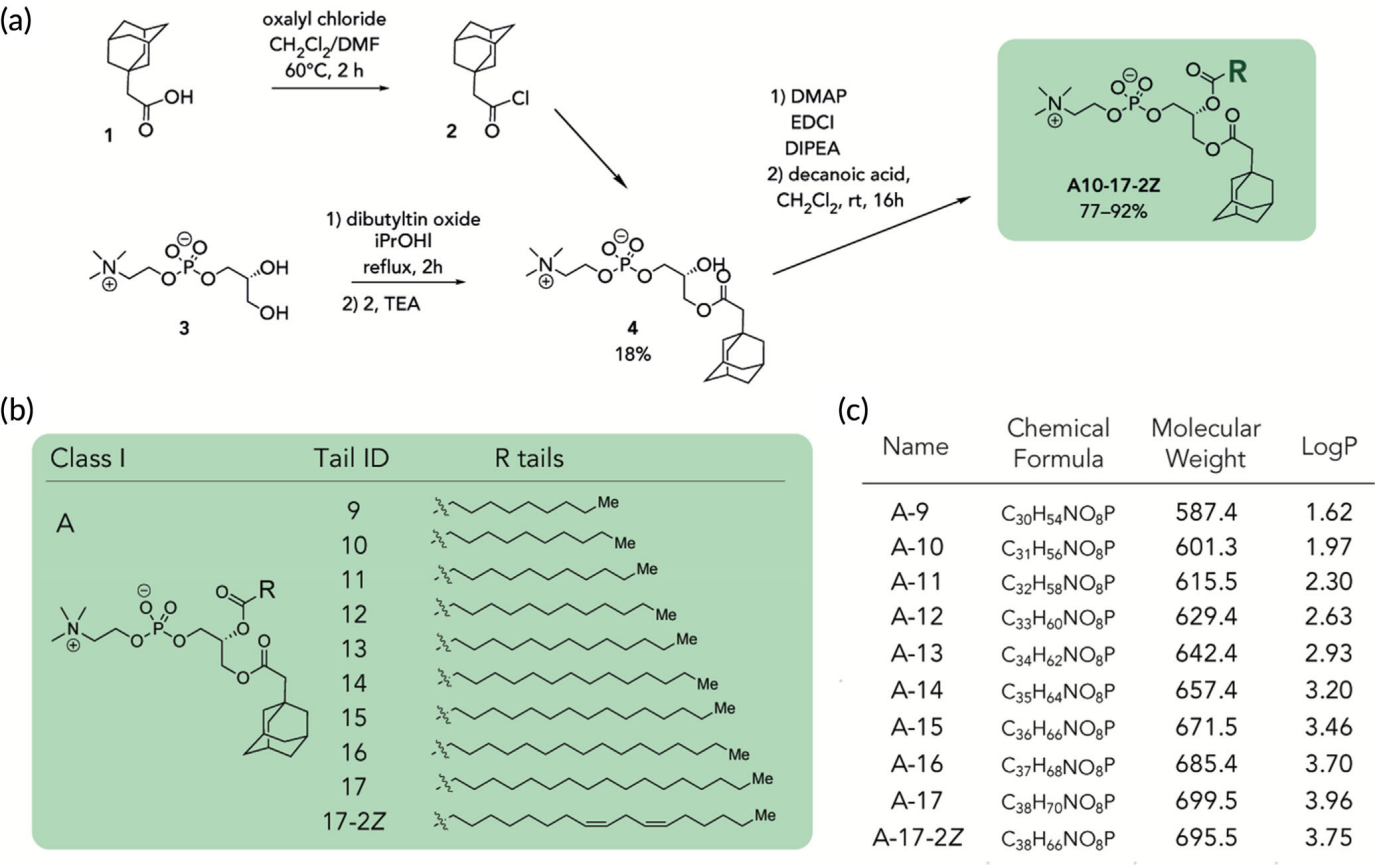
Synthesis of phospholipids with a constrained adamantyl group. (a) Synthesis schematic for the production of constrained phospholipids. (b) Constrained phospholipids with 10 variable hydrocarbon chains have a (c) variety of chemical properties

We first synthesized a series of novel constrained phospholipids (Figure 1a). Each phospholipid contained a terminal quaternary ammonium headgroup, phosphodiester linkage, and two hydrocarbon chains added via esterification. One of the hydrocarbon chains was an adamantyl, while the second chain was unconstrained; we varied the length and saturation of the second chain based on evidence that altering these variables in cationic lipids affects how nanoparticles interact with the cell bilayer, and potentially, endosomal membranes.^22^ We did not synthesize compounds with two adamantyl groups since early data suggested ionizable amines containing two adamantyl groups may lose activity in vivo.^15^ To synthesize the phospholipids, we applied a selective mono‐acylation of l‐*α*‐glycerylphosphorylcholine**3**, which enabled the preparation of our target phospholipid in two steps (Figure 1a). In the first step, we synthesized 1‐substituted‐sn‐glycero‐3‐phosphocholine**4** via dibutyltin oxide‐mediated selective primary alcohol acylation of 1,2‐diols with adamantyl chloride **2**.^23^ Finally, Steglich esterification^24^ of variable hydrocarbon tail to the remaining hydroxyl group afforded 10 compounds, **A‐10** to **A‐17‐2Z**(Figure 1b,c) in 77–92% of yields. We characterized these compounds by ^1^H‐NMR, ^13^C‐NMR, and high‐resolution mass spectrometry (see Material and Methods), and for simplicity, termed these “constrained phospholipids”.

**FIGURE 2:**
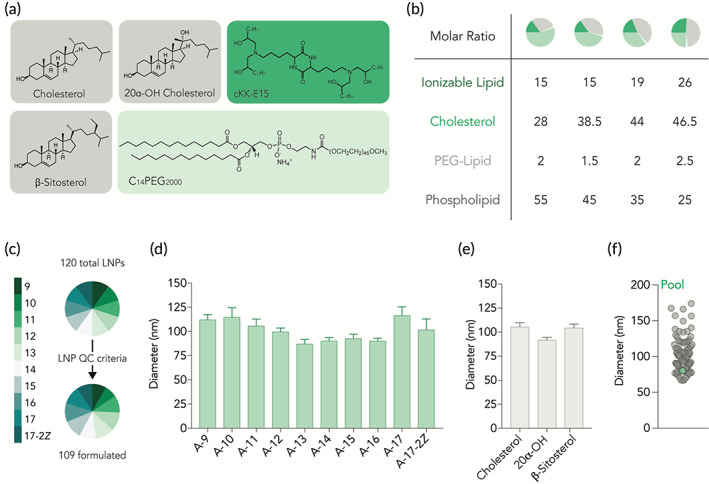
Constrained phospholipids can be used to form stable LNPs. 120 LNPs were formulated by combining constrained phospholipids with (a) three sterol variants, C14PEG2000, and cKK‐E15 at (b) four different formulation ratios. (c) 109 LNPs passed quality control criteria and had a diameter less than 200 nm as well as a stable autocorrelation curve. LNP diameter was not impacted by the presence of (d) different hydrocarbon tails or (e) sterol variant used. (f) The average diameter of the 109 LNP pool administered to mice was within range of the individual LNPs that make up the pool

We then investigated whether these novel constrained phospholipids were capable of forming stable LNPs. To isolate the effect of constrained phospholipids on LNP formation, we used a lipid‐PEG(C_14_PEG_2000_), cholesterol (cholesterol, 20α‐hydroxycholesterol, β‐sitosterol), and ionizable lipid (cKK‐E15) components that were previously characterized (Figure 2a), and utilized four different molar ratios (Figure 2b). In order to perform a high throughput in vivo study, we utilized Fast Identification of Nanoparticle Delivery^20^(FIND), a workflow^18^, ^19^, ^20^ that quantifies mRNA delivery mediated by many LNPs in a single mouse. Briefly, we used microfluidics to formulate 120 LNPs with Cre mRNA and a unique DNA barcode; LNP‐1, with chemical structure 1, carried Cre mRNA and DNA barcode 1, whereas LNP‐N, with chemical structure N, carried Cre mRNA and DNA barcode N. We performed a quality control analysis on all 120 LNPs by quantifying the size of each LNP using dynamic light scattering. We only included LNPs that were monodisperse and had a hydrodynamic diameter less than 200 nm. Of the 120 formulated LNPs, 109 met these quality control criteria, and were pooled together (Figure 2c).

To evaluate whether constrained phospholipids consistently formed LNPs, we quantified the average hydrodynamic diameter of each LNP that met quality control criteria as a function of phospholipid structure; constrained phospholipids consistently formed small LNPs (Figure 2d, Figure S1a). Similarly, we then investigated whether phospholipids formed stable LNPs independent of cholesterol structure and found this to be true (Figure 2e, Figure S1a). Finally, as a control, we plotted the average diameter of the 109 LNP pool and found that it was within range of the 109 individual LNPs making it up (Figure 2f). Taken together, these data led us to conclude that stable LNPs can be formulated using constrained phospholipids, and that these LNPs did not “crash out” of solution when mixed together.

**FIGURE 3:**
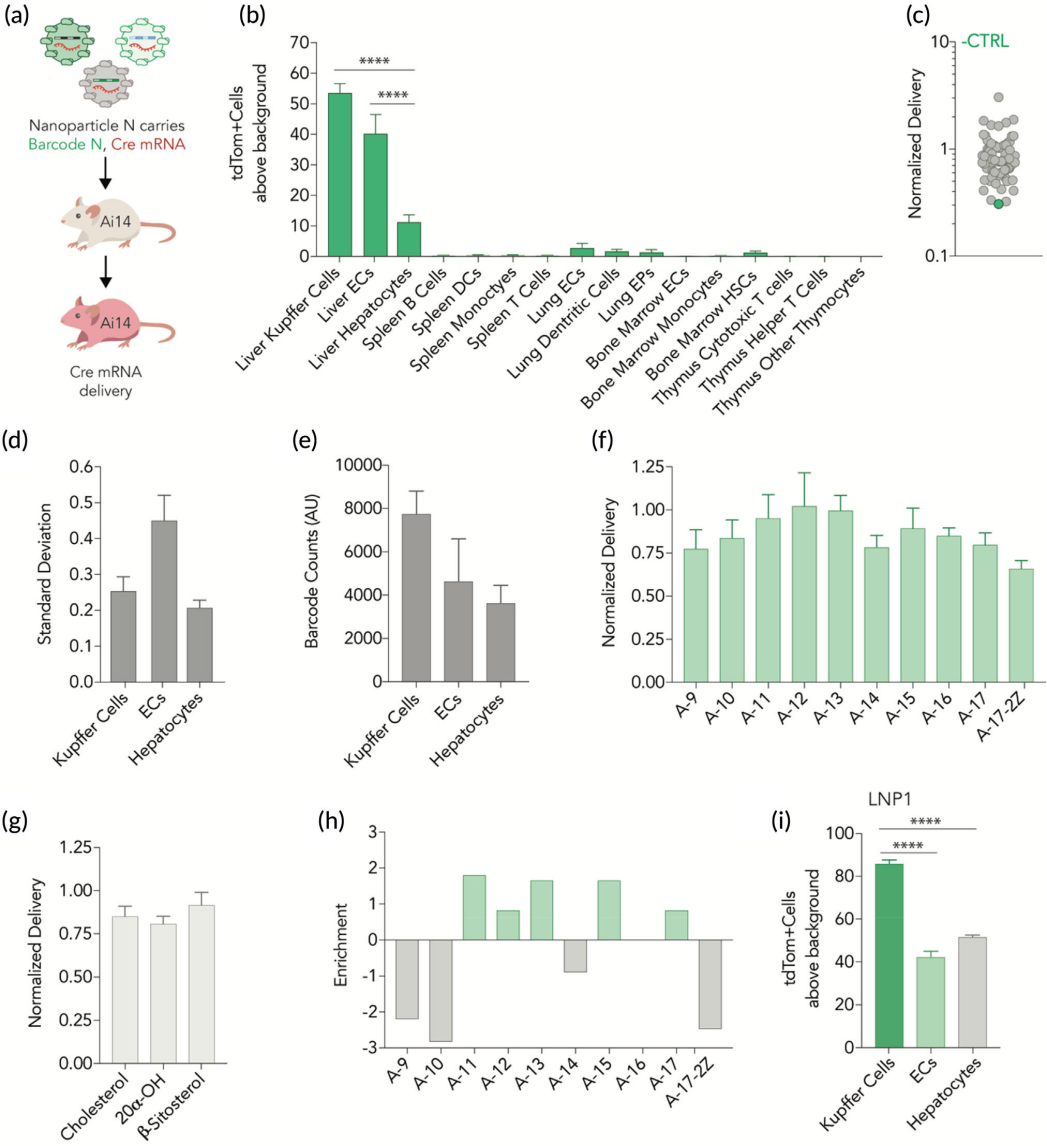
LNPs containing constrained phospholipids deliver mRNA to non‐hepatocytecell‐types. (a) LNPs were formulated to carry a unique DNA barcode and Cre mRNA. The 109 LNP pool was then administered to Ai14 mice. After 3 days tdTomato signal was quantified in (b) multiple cell types in the liver, spleen, lung, bone marrow, and thymus. Kupffer cells and liver endothelial cells were preferentially targeted. Cell types with tdTomato signal were sorted and sequenced. (c) The negative control (free DNA barcode) was delivered into cells less efficiently than barcodes carried in LNPs, measured across all cell types. Similarly, (d) standard deviations for normalized delivery across liver cell types was comparable. (e) Kupffer cells had the highest LNP biodistribution, relative to liver endothelial cells and hepatocytes. Average normalized delivery across all cell types was not impacted by (f) changes in the hydrocarbon tail of constrained phospholipids or (g) the sterol variant. (h) Fold enrichment identified five constrained phospholipids that perform well in the top 10% of LNPs. This analysis helped identify an LNP containing A‐11(LNP1) which (i) preferentially delivers mRNA to kupffer cells in the liver over both endothelial cells and hepatocytes. ****p < .0001, two‐way ANOVA

We then analyzed whether the 109 LNPs delivered mRNA in vivo using FIND.^20^ We intravenously injected the LNP pool containing Cre mRNA and a DNA barcode into Ai14 mice at a total nucleic acid dose of 1.0 mg/kg (0.009 mg/kg/particle, on average). Ai14 mice have a Lox‐Stop‐Lox‐tdTomato construct downstream a CAG promoter; cells become tdTomato^+^ only if Cre mRNA is delivered into the cytoplasm and translated into functional Cre protein that edits the Stop site out of the genome (Figure 3a). By isolating tdTomato^+^ cells using fluorescence activated cell sorting (FACS), and quantifying the barcodes in those cells using next‐generation DNA sequencing, we identified barcodes in cells that were transfected by functional mRNA.^18^, ^19^, ^20^ We quantified the percentage of cells in which Cre mRNA had been functionally delivered (i.e., the tdTomato^+^ cells) from 16 cell populations (Figure S1b) and found that constrained phospholipids delivered mRNA to Kupffer cells and endothelial cells (ECs) efficiently (Figure 3b). We then evaluated three additional controls. First, we quantified the amount of free barcode; we included this as a negative control, since DNA does not readily enter cells on its own. As expected, free barcode was delivered into cells less efficiently than barcodes encapsulated by LNPs (Figure 3c). Second, we found that the LNP sequencing data was consistent across mouse replicates (Figure 3d). Third, we quantified mouse weight. Relative to PBS‐treated mice, those treated with barcodes did not lose weight (Figure S1c).

Based on these controls, we concluded it was appropriate to perform additional analyses, in order to further understand the observed non‐hepatocyte tropism. We investigated whether the amount of LNPs that reached the cells (i.e., biodistribution) mirrored the functional readouts. We therefore measured the LNP biodistribution of the 109 LNP pool across liver cell‐types using QUANT, a technique used to quantify LNP biodistribution using droplet digital PCR (ddPCR)^25^; we previously compared QUANT biodistribution readouts to fluorescent biodistribution readouts and found QUANT to be more sensitive.^25^ After isolating the tdTomato^+^ liver cells using FACS, we used ddPCR to quantify the absolute amount of DNA barcode in Kupffer cells, liver endothelial cells, and hepatocytes. The biodistribution profile of the LNP pool was similar to the functional delivery profile (Figure 3e).

We then used next‐generation sequencing to analyze how each of the 109 individual LNPs delivered nucleic acids. We plotted the normalized delivery, that is, the amount of individual barcode N divided by the sum of all barcodes in that cell type, for all N barcodes.^26^ When we plotted normalized delivery as a function of the phospholipid (Figure 3f) or cholesterol (Figure 3g) structure, we did not observe substantial differences. We subsequently calculated the enrichment of different nanoparticle properties.^18^ Briefly, we calculated how often a property would appear in particles that performed in the top 10% and particles that performed in the bottom 10%. We then calculated fold enrichment change by subtracting enrichment in the bottom 10% from enrichment in the top 10%. We found phospholipid A‐11 was most highly enriched in Kupffer cells, liver ECs, and hepatocytes (Figure 3h). Based on these screening data, we selected an individual LNP formulated with phospholipid A‐11, and quantified whether it delivered mRNA as predicted by the screen. Specifically, we formulated this LNP (termed LNP1 for simplicity) to carry Cre mRNA and injected Ai14 mice intravenously at a dose of 0.5 mg/kg. Three days later, we quantified the percentage of tdTomato^+^ cells, and found liver Kupffer cells were preferentially targeted, as predicted by the screen (Figure 3i). LNP1 did not lead to any tdTomato^+^ cells in spleen T cells, B cells, or monocytes (Figure S2a), which are common off‐target cell types. Relative to PBS‐treated mice, those treated with LNP1 did not lose weight (Figure S2b). Taken together, these data led us to conclude that LNPs with adamantyl‐based phospholipids can deliver mRNA to non‐hepatocyte cell types in vivo.

## 3 MATERIALS AND METHODS

### 3.1 General methods

Starting materials, reagents and solvents were purchased from commercial sources and used as received unless stated otherwise. Purification of reactions products was performed by column chromatography using silica gel (300‐400 mesh) and eluting with hexane/ethyl acetate, DCM/MeOH. Thin layer chromatography (TLC) was carried out using precoated silica Gel GF plates and visualized using KMnO_4_ stains. ^1^H‐NMR spectra were recorded at 400 or 500 MHz (Varian) using CDCl_3_ with TMS. High‐resolution mass spectra (HRMS) were recorded on LC/MS (Agilent Technologies 1260 Infinity II/6120 Quadrupole) and a time‐of‐flight mass spectrometer by ESI or matrix assisted laser desorption/ionization (MALDI).

### 3.2 Phospholipids Synthesis

2‐((3*r*,5*r*,7*r*)‐Adamantan‐1‐yl)acetyl chloride (**2**)^1^

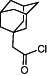

To a solution of 1‐adamantaneacetic acid **1** (2.0 g, 10.3 mmol, 1 eq) and *N*,*N*‐dimethylformamide (DMF) (0.1 mL, 0.1 mmol) in anhydrous DCM (30 mL), under nitrogen atmosphere, was added oxalyl chloride (2.6 mL, 30.9 mmol, 3 eq.) at 25 °C. The reaction mixture was refluxed at 60 ºC for 2 h and then concentrated under reduced pressure to provide crude product **2** as yellow oil (2.1 g, 95%), which was directly used in the next step without further purification.

(*R*)‐3‐(2‐((3*R*,5*R*,7*R*)‐Adamantan‐1‐yl)acetoxy)‐2‐hydroxypropyl(2‐(trimethylammonio)ethyl) phosphate (**4**)

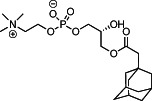

*sn*‐Glycero‐3‐phosphocholine **3** (1.3 g, 5.0 mmol, 1 eq) and dibutyltin oxide (1.3 g, 5.5 mmol, 1.1 eq) were suspended in isopropanol (40 mL). The reaction mixture was refluxed for 2 h, then cooled down to 25 °C and treated with triethylamine (0.75 mL, 5.5 mmol, 1.1 eq). 2‐((3*r*,5*r*,7*r*)‐adamantan‐1‐yl)acetyl chloride **2** was dissolved in DCM (2 mL) and added to the reaction under nitrogen atmosphere. The reaction mixture was stirred for 1 h and then concentrated under reduced pressure. The resulting residue was purified by flash chromatography (silica gel, gradient eluent: 10‐20% of MeOH/DCM then 20% of MeOH/DCM containing with 1% ammonium hydroxide then 50% of MeOH/DCM containing with 10% ammonium hydroxide) to provide the desired product **4** (400 mg, 18 % yield) as colorless oil.

^**1**^ **H NMR** (400 MHz, CDCl_3_/MeOD): δ 1.47 (br. s, 10H), 1.56 (br. s, 3H), 1.83 (s, 2H), 1.96 (t, *J* = 4.8 Hz, 2H), 3.08 (s, 3H), 3.48 (s, 2H), 3.71–3.74 (m, 1H), 3.83–3.84 (m, 1H), 3.96 (s, 3H), 4.13 (s, 2H).

^**13**^ **C NMR** (100 MHz, CDCl_3_/MeOD): δ 28.3, 32.5, 36.4, 42.1, 53.9, 58.8, 64.4, 66.2, 66.8, 68.6, 171.9.

**HRMS** (ESI, m/z) calcd for C_20_H_36_NNaO_7_P [M + Na]^+^: 456.2122, found 456.2118.

(*R*)‐3‐(2‐((3*R*,5*R*,7*R*)‐Adamantan‐1‐yl)acetoxy)‐2‐(decanoyloxy)propyl(2‐(trimethylammonio)ethyl) phosphate (**A‐9**)

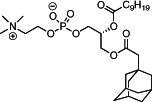

To a solution of product **4** (30 mg, 0.07 mmol, 1 eq), DMAP (3.7 mg, 0. 03 mmol, 0.4 eq), DIPEA (50.0 μL, 0.28 mmol, 4 eq) and decanoic acid (48 mg, 0.28 mmol, 4 eq) in anhydrous DCM (2 mL) was added *N*‐(3‐dimethylaminopropyl)‐*N*'‐ethylcarbodiimide hydrochloride (EDCI) (53 mg, 0.28 mmol, 4 eq) under nitrogen atmosphere. The reaction mixture was stirred at 25 °C for 16 h and then concentrated under reduced pressure. The resulting residue was purified by flash chromatography (silica gel, gradient eluent: 10‐50% of MeOH/DCM then 50% of MeOH/DCM containing with 1‐10% ammonium hydroxide) to provide the desired product **A‐9** (38 mg, 92% yield) as colorless oil.

^**1**^ **H NMR** (500 MHz, CDCl_3_): δ 0.87 (t, *J* = 7.5 Hz, 3H), 1.25 (br. s, 14H), 1.57 (s, 6H), 1.59–1.70 (m, 7H), 1.95 (s, 2H), 2.05 (s, 2H), 2.28 (td, *J* = 7.5, 3.5 Hz, 2H), 3.29 (s, 9H), 3.71 (br. s, 2H), 3.86–3.97 (m, 2H), 4.09–4.12 (m, 1H), 4.26 (br. s, 2H), 4.37 (dd, *J* = 12.0, 2.0 Hz, 1H), 5.17 (br. s, 1H).

^**13**^ **C NMR** (125 MHz, CDCl_3_): δ 14.1, 22.7, 24.9, 28.5, 29.2, 29.3, 29.4, 29.5, 31.9, 32.7, 34.3, 36.7, 42.3, 48.8, 54.3, 59.4, 62.7, 63.5, 66.2, 70.5, 171.5, 173.2.

**HRMS** (ESI, m/z) calcd for C_30_H_54_NNaO_8_P [M + Na]^+^: 610.3479, found 610.3468.

(*R*)‐2‐(2‐((3*R*,5*R*,7*R*)‐Adamantan‐1‐yl)acetoxy)‐3‐(undecanoyloxy)propyl(2‐(trimethylammonio)ethyl) phosphate (**A‐10**)

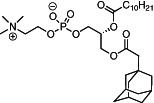

Product **A‐10** was synthesized following the same procedure as product **A‐9** using compound **4** (30 mg, 0.07 mmol, 1 eq), DMAP (3.7 mg, 0. 03 mmol, 0.4 eq), DIPEA (50.0 μL, 0.28 mmol, 4 eq) and undecanoic acid (52 mg, 0.28 mmol, 4 eq) in anhydrous CH_2_Cl_2_ (2 mL) was added EDCI (53 mg, 0.28 mmol, 4 eq) under nitrogen atmosphere and stirred overnight at 25 °C to provide **A‐10** (34 mg, 78% yield) as a colorless oil.

^**1**^ **H NMR** (400 MHz, CDCl_3_) δ 0.87 (t, *J* = 7.2 Hz, 3H), 1.25 (br. s, 16H), 1.57 (s, 6H), 1.57‐1.70 (m, 7H), 1.94 (s, 2H), 2.05 (s, 2H), 2.28 (t, *J* = 7.2 Hz, 2H), 3.29 (s, 9H), 3.71 (br. s, 2H), 3.89‐3.91 (m, 2H), 4.08–4.13 (m, 2H), 4.25 (br. s, 2H), 4.37 (dd, *J* = 12.0, 2.4 Hz, 1H), 5.17‐5.18 (m, 1H).

^**13**^ **C NMR** (100 MHz, CDCl_3_) δ 14.1, 22.7, 24.9, 28.6, 29.2, 29.3, 29.4, 29.6, 31.9, 32.7, 34.3, 36.7, 42.3, 48.8, 54.3, 59.4, 62.7, 63.4, 66.3, 70.5, 171.5, 173.2.

**HRMS** (ESI, m/z) calcd for C_31_H_56_NNaO_8_P [M + Na]^+^: 624.3636, found 624.3624.

(*R*)‐2‐(2‐((3*R*,5*R*,7*R*)‐Adamantan‐1‐yl)acetoxy)‐3‐(dodecanoyloxy)propyl(2‐(trimethylammonio)ethyl) phosphate (**A‐11**)

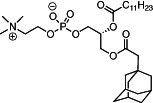

Product **A‐11** was synthesized following the same procedure as product **A‐9** using compound **4** (30 mg, 0.07 mmol, 1 eq), DMAP (3.7 mg, 0. 03 mmol, 0.4 eq), DIPEA (50.0 μL, 0.28 mmol, 4 eq) and lauric acid (56 mg, 0.28 mmol, 4 eq) in anhydrous CH_2_Cl_2_ (2 mL) was added EDCI (53 mg, 0.28 mmol, 4 eq) under nitrogen atmosphere and stirred overnight at 25 °C to provide **A‐11** (38 mg, 85% yield) as a colorless oil.

^**1**^ **H NMR** (400 MHz, CDCl_3_) δ 0.87 (t, *J* = 7.2 Hz, 3H), 1.25 (br. s, 18H), 1.56 (br. s, 6H), 1.59‐1.70 (m, 7H), 1.94 (s, 2H), 2.04 (s, 2H), 2.28 (td, *J* = 7.2, 2.0 Hz, 2H), 3.29 (s, 9H), 3.71 (br. s, 2H), 3.87‐3.95 (m, 2H), 4.08–4.13 (m, 1H), 4.26 (br. s, 2H), 4.37 (dd, *J* = 12.0, 2.4 Hz, 1H), 5.16‐5.18 (m, 1H).

^**13**^ **C NMR** (100 MHz, CDCl_3_) δ 14.1, 22.7, 24.9, 28.6, 29.3, 29.4, 29.5, 29.7, 29.8, 31.9, 32.7, 34.3, 36.7, 42.3, 48.7, 54.2, 59.4, 62.8, 63.6, 66.2, 70.5, 171.8, 173.5.

**HRMS** (ESI, m/z) calcd for C_32_H_58_NNaO_8_P [M + Na]^+^: 638.3792, found 638.3780.

(*R*)‐3‐(2‐((3*R*,5*R*,7*R*)‐Adamantan‐1‐yl)acetoxy)‐2‐(tridecanoyloxy)propyl (2‐(trimethylammonio)ethyl) phosphate (**A‐12**)

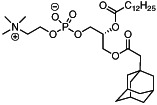

Product **A‐12** was synthesized following the same procedure as product **A‐9** using compound **4** (30 mg, 0.07 mmol, 1 eq), DMAP (3.7 mg, 0. 03 mmol, 0.4 eq), DIPEA (50.0 μL, 0.28 mmol, 4 eq) and tridecanoic acid (60 mg, 0.28 mmol, 4 eq) in anhydrous CH_2_Cl_2_ (2 mL) was added EDCI (53 mg, 0.28 mmol, 4 eq) under nitrogen atmosphere and stirred overnight at 25 °C to provide **A‐12** (35 mg, 77% yield) as a colorless oil.

^**1**^ **H NMR** (400 MHz, CDCl_3_) δ 0.87 (t, *J* = 6.8 Hz, 3H), 1.24 (br. s, 20H), 1.56 (br. s, 6H), 1.59‐1.70 (m, 7H), 1.94 (br. s, 2H), 2.05 (s, 2H), 2.29 (td, *J* = 7.6 , 2.0 Hz, 2H), 3.29 (s, 9H), 3.71 (br. s, 2H), 3.89‐3.91 (m, 2H), 4.08–4.13 (m, 1H), 4.25 (br. s, 2H), 4.37 (dd, *J* = 12.0, 2.4 Hz, 1H), 5.13‐5.21 (m, 1H).

^**13**^ **C NMR** (100 MHz, CDCl_3_) δ 14.1, 22.7, 24.9, 28.6, 29.2, 29.3, 29.4, 29.5, 29.6, 29.6, 29.7, 31.9, 32.7, 34.3, 36.7, 42.3, 48.8, 54.3, 59.4, 62.7, 63.4, 66.3, 70.5, 171.5, 173.2.

**HRMS** (ESI, m/z) calcd for C_33_H_60_NNaO_8_P [M + Na]^+^: 652.3949, found 652.3939.

(*R*)‐3‐(2‐((3*R*,5*R*,7*R*)‐Adamantan‐1‐yl)acetoxy)‐2‐(tetradecanoyloxy)propyl(2(trimethylammonio)ethyl) phosphate (**A‐13**)

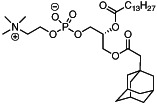

Product **A‐13** was synthesized following the same procedure as product **A‐9** using compound **4** (30 mg, 0.07 mmol, 4 eq), DMAP (3.7 mg, 0. 03 mmol, 0.4 eq), DIPEA (50.0 μL, 0.28 mmol, 4 eq) and myristic acid (64 mg, 0.28 mmol, 4 eq) in anhydrous CH_2_Cl_2_ (2 mL) under nitrogen atmosphere was added EDCI (53 mg, 0.28 mmol, 4 eq) under nitrogen atmosphere and stirred overnight at 25 °C to provide **A‐13** (39 mg, 84% yield) as a colorless oil.

^**1**^ **H NMR** (400 MHz, CDCl_3_) δ 0.87 (t, *J* = 7.2 Hz, 3H), 1.24‐1.30 (m, 22H), 1.57 (br. s, 6H), 1.59‐1.70 (m, 7H), 1.94 (br. s, 2H), 2.05 (s, 2H), 2.28 (td, *J* = 7.5, 2.4 Hz, 2H), 3.30 (s, 9H), 3.72‐3.74 (m, 2H), 3.88‐3.93 (m, 2H), 4.08–4.13 (m, 1H), 4.25‐4.26 (m, 2H), 4.37 (dd, *J* = 12.0, 2.4 Hz, 1H), 5.17‐5.18 (m, 1H).

^**13**^ **C NMR** (100 MHz, CDCl_3_) δ 14.1, 22.7, 24.9, 28.6, 29.2, 29.3, 29.4, 29.6, 29.6, 29.7, 29.7, 31.9, 32.7, 34.3, 36.7, 42.3, 48.8, 54.3, 59.4, 62.7, 63.5, 66.2, 70.5, 171.5, 173.2.

**HRMS** (ESI, m/z) calcd for C_34_H_63_NO_8_P [M + H]^+^: 644.4286, found 644.4278.

(*R*)‐3‐(2‐((3*R*,5*R*,7*R*)‐Adamantan‐1‐yl)acetoxy)‐2‐(pentadecanoyloxy)propyl(2(trimethyl‐‐ammonio)ethyl) phosphate (**A‐14**)

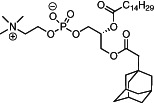

Product **A‐14** was synthesized following the same procedure as product **A‐9** using compound **4** (30 mg, 0.07 mmol, 1 eq), DMAP (3.7 mg, 0. 03 mmol, 0.4 eq), DIPEA (50.0 μL, 0.28 mmol, 4 eq) and pentadecanoic acid (68 mg, 0.28 mmol, 4 eq) in anhydrous CH_2_Cl_2_ (2 mL) was added EDCI (53 mg, 0.28 mmol, 4 eq) under nitrogen atmosphere and stirred overnight at 25 °C to provide **A‐14** (39 mg, 82% yield) as a colorless oil.

^**1**^ **H NMR** (500 MHz, CDCl_3_) δ 0.87 (t, *J* = 7.0 Hz, 3H), 1.24 (br. s, 24H), 1.57 (br. s, 6H), 1.59‐1.70 (m, 7H), 1.94 (br. s, 2H), 2.04 (s, 2H), 2.26–2.30 (m, 2H), 3.29 (s, 9H), 3.72 (br. s, 2H), 3.89‐3.93 (m, 2H), 4.08–4.12 (m, 1H), 4.25 (m, 2H), 4.37 (dd, *J* = 12.0, 2.0 Hz, 1H), 5.17‐5.18 (m, 1H).

^**13**^ **C NMR** (125 MHz, CDCl_3_) δ 14.1, 22.7, 24.9, 28.5, 29.2, 29.3, 29.4, 29.6, 29.6, 29.6, 29.7, 31.9, 32.7, 34.3, 36.7, 42.3, 48.7, 54.2, 59.4, 62.7, 63.5, 66.2, 70.5, 171.5, 173.2.

**HRMS** (ESI, m/z) calcd for C_35_H_64_NNaO_8_P [M + Na]^+^: 680.4262, found 680.4253.

(*R*)‐3‐(2‐((3*R*,5*R*,7*R*)‐Adamantan‐1‐yl)acetoxy)‐2‐(palmitoyloxy)propyl (2‐(trimethylammonio)ethyl) phosphate (**A‐15**)

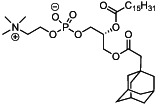

Product **A‐15** was synthesized following the same procedure as product **A‐9** using compound **4** (30 mg, 0.07 mmol, 1 eq), DMAP (3.7 mg, 0. 03 mmol, 0.4 eq), DIPEA (50.0 μL, 0.28 mmol, 4 eq) and palmitic acid (72 mg, 0.28 mmol, 4 eq) in anhydrous CH_2_Cl_2_ (2 mL) was added EDCI (53 mg, 0.28 mmol, 4 eq) under nitrogen atmosphere and stirred overnight at 25 °C to provide **A‐15** (43 mg, 89% yield) as a colorless oil.

^**1**^ **H NMR** (500 MHz, CDCl_3_) δ 0.87 (t, *J* = 7.0 Hz, 3H), 1.24 (br. s, 26H), 1.57 (s, 6H), 1.67‐1.70 (m, 7H), 1.94 (s, 2H), 2.05 (s, 2H), 2.26–2.30 (m, 2H), 3.28 (s, 9H), 3.70 (s, 2H), 3.89–3.92 (m, 2H), 4.08–4.12 (m, 1H), 4.25–4.38 (m, 2H), 5.17 (br. s, 1H).

^**13**^ **C NMR** (125 MHz, CDCl_3_) δ 14.1, 22.7, 24.9, 28.5, 29.2, 29.3, 29.4, 29.6 (two carbons overlapped each other), 29.7 (two carbons overlapped each other), 31.9, 32.7, 34.3, 36.7, 42.3, 48.8, 54.2, 59.4, 62.7, 63.5, 66.2, 70.5, 171.5, 173.2.

**HRMS** (ESI, m/z) calcd for C_36_H_66_NNaO_8_P [M + Na]^+^: 694.4418, found 694.4417.

(R)‐3‐(2‐((3R,5R,7R)‐Adamantan‐1‐yl)acetoxy)‐2‐(heptadecanoyloxy)propyl(2‐(trimethyl‐‐ammonio)ethyl) phosphate (**A‐16**)

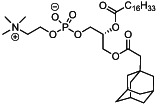

Product **A‐16** was synthesized following the same procedure as product **A‐9** using compound **4** (30 mg, 0.07 mmol, 1 eq), DMAP (3.7 mg, 0. 03 mmol, 0.4 eq), DIPEA (50.0 μL, 0.28 mmol, 4 eq) and heptadecanoic acid (76 mg, 0.28 mmol, 4 eq) in anhydrous CH_2_Cl_2_ (2 mL) was added EDCI (53 mg, 0.28 mmol, 4 eq) under nitrogen atmosphere and stirred overnight at 25 °C to provide **A‐16** (38 mg, 77% yield) as a colorless oil.

^**1**^ **H NMR** (400 MHz, CDCl_3_) δ 0.87 (t, *J* = 7.0 Hz, 3H), 1.24 (br. s, 28H), 1.57 (s, 6H), 1.59‐1.67 (m, 7H), 1.94 (s, 2H), 2.05 (s, 2H), 2.28–2.31 (m, 2H), 3.30 (s, 9H), 3.73 (s, 2H), 3.92 (br. s, 2H), 4.10–4.11 (m, 1H), 4.35–4.38 (m, 2H), 5.17 (br. s, 1H).

^**13**^ **C NMR** (100 MHz, CDCl_3_) δ 14.1, 22.7, 24.9, 28.6, 29.2, 29.3, 29.4, 29.6 (two carbons overlapped each other), 29.7 (three carbons overlapped each other), 31.9, 32.7, 34.3, 36.7, 42.3, 48.8, 54.3, 59.5, 62.7, 63.5, 66.2, 70.5, 171.5, 173.2.

**HRMS** (ESI, m/z) calcd for C_37_H_68_NO_8_P [M + Na]^+^: 708.4575, found 708.4565.

(R)‐3‐(2‐((3R,5R,7R)‐Adamantan‐1‐yl)acetoxy)‐2‐(stearoyloxy)propyl (2‐(trimethylammonio)ethyl) phosphate (**A‐17**)

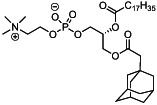

Product **A‐17** was synthesized following the same procedure as product **A‐9** using compound **4** (30 mg, 0.07 mmol, 1 eq), DMAP (3.7 mg, 0. 03 mmol, 0.4 eq), DIPEA (50.0 μL, 0.28 mmol, 4 eq) and stearic acid (79 mg, 0.28 mmol, 4 eq) in anhydrous CH_2_Cl_2_ (2 mL) was added EDCI (53 mg, 0.28 mmol, 4 eq) under nitrogen atmosphere and stirred overnight at 25 °C to provide **A‐17** (39 mg, 77% yield) as a colorless oil.

^**1**^ **H NMR** (400 MHz, CDCl_3_) δ 0.87 (t, *J* = 7.2 Hz, 3H), 1.24 (br. s, 30H), 1.57 (s, 6H), 1.59‐1.70 (m, 7H), 1.95 (s, 2H), 2.05 (s, 2H), 2.26–2.31 (m, 2H), 3.30 (s, 9H), 3.73 (s, 2H), 3.91–3.92 (m, 2H), 4.10–4.27 (m, 1H), 4.35–4.39 (m, 2H), 5.17–5.18 (m, 1H).

^**13**^ **C NMR** (100 MHz, CDCl_3_) δ 14.1, 22.7, 25.0, 28.6, 29.3, 29.4 (two carbons overlapped each other), 29.6, 29.7 (two carbons overlapped each other), 29.8, 31.9, 32.7, 34.4, 36.7, 42.3, 48.8, 54.3, 59.4, 62.7, 63.5, 66.3, 70.5, 171.5, 173.2.

**HRMS** (ESI, m/z) calcd for C_38_H_70_NNaO_8_P [M + Na]^+^: 722.4731, found 722.4723.

(R)‐3‐(2‐((3R,5R,7R)‐Adamantan‐1‐yl)acetoxy)‐2‐(((9Z,12Z)‐octadeca‐9,12‐dienoyl)oxy)propyl (2‐(trimethylammonio)ethyl) phosphate (**A‐17‐2Z**)

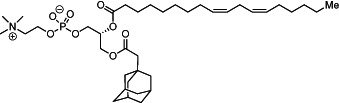

Product **A‐17‐2Z** was synthesized following the same procedure as product **A‐9** using compound **4** (30 mg, 0.07 mmol, 1 eq), DMAP (3.7 mg, 0. 03 mmol, 0.4 eq), DIPEA (50.0 μL, 0.28 mmol, 4 eq) and linoleic acid (79 mg, 0.28 mmol, 4 eq) in anhydrous CH_2_Cl_2_ (2 mL) was added EDCI (53 mg, 0.28 mmol, 4 eq.) under nitrogen atmosphere and stirred overnight at 25 °C to provide **A‐17‐2Z** (41 mg, 82% yield) as a colorless oil.

^**1**^ **H NMR** (400 MHz, CDCl_3_) δ 0.87 (t, *J* = 7.2 Hz, 3H), 1.28 (br. s, 16H), 1.57 (s, 6H), 1.59‐1.70 (m, 7H), 1.94 (s, 2H), 1.99‐2.04 (m, 7H), 2.26‐2.30 (m, 2H), 2.75 (t, *J* = 6.8 Hz, 1H), 3.29 (s, 9H), 3.70 (br. s, 2H), 3.84‐3.97 (m, 2H), 4.08–4.13 (m, 1H), 4.25 (s, 2H), 4.36–4.39 (m, 1H), 5.13‐5.21 (m, 1H), 5.26‐5.43 (m, 3H).

^**13**^ **C NMR** (100 MHz, CDCl_3_) δ 14.0, 22.5, 24.9, 25.6, 27.2, 28.6, 29.2, 29.3, 29.5, 29.7, 29.8, 31.5, 31.9, 32.7, 34.3, 36.7, 42.3, 48.8, 54.3, 59.4, 62.7, 63.5, 66.2, 70.5, 127.8, 128.0, 130.0, 130.2, 171.5, 173.1.

**HRMS** (ESI, m/z) calcd for C_38_H_66_NNaO_8_P [M + Na]^+^: 718.4418, found 718.4399.

### 3.3 Nanoparticle Formulation

Nanoparticles were formulated with a NanoAssemblr Spark Formulation Device (Precision Nanosystems). Briefly, nucleic acids (DNA barcodes, mRNA, and sgRNA) were diluted in 10mM citrate buffer (Teknova). Lipid‐amine compounds, alkyl‐tailed PEG, cholesterol, and helper lipids were diluted in 100% ethanol. Both phases were loaded into the NanoAssemblr Spark Cartridge. An optimized protocol, designed by Precision Nanosystems for the NanoAssemblr Spark, was followed. For nanoparticle screens, Cre mRNA and DNA barcodes were mixed at a 10:1 mass ratio. All PEGs and cholesterol were purchased from Avanti Lipids.

### 3.4 DNA Barcoding

Each LNP was formulated to carry a unique DNA barcode. LNP1 carried DNA barcode 1, while the chemically distinct LNPN carried DNA barcode N. DNA barcodes were designed rationally with several characteristics, as we previously described^2,3^. Single stranded DNA barcodes were purchased from Integrated DNA Technologies. Each barcode was distinguished using a unique 8 nucleotide sequence. An 8‐nucleotide sequence can generate over 4^8^ (65,536) distinct barcodes. We used distinct 8 nucleotide sequences designed by to prevent sequence bleaching and reading errors on the Illumina MiniSeq^TM^ sequencing machine.

### 3.5 Nanoparticle Characterization

LNP hydrodynamic diameter was measured using high throughput dynamic light scattering (DLS) (DynaPro Plate Reader II, Wyatt). LNPs were diluted in sterile 1X PBS and analyzed. To avoid using unstable LNPs, and to enable sterile purification using a 0.22 μm filter, LNPs were included only if they met 3 criteria: diameter >20 nm, diameter <200 nm, and correlation function with 1 inflection point. Particles that met these criteria were dialyzed with 1X PBS.

### 3.6 Animal Experiments

All animal experiments were performed in accordance with the Georgia Institute of Technology’s IACUC. All animals were bread in the Georgia Institute of Technology Animal Facility. C57BL/6J (#000664) were purchased from The Jackson Laboratory. LSL‐Tomato/Ai14 (#007914) were purchased from The Jackson Laboratory for breeding proposes. In all experiments, we used N=3‐5 mice / group. Mice were injected intravenously via the lateral tail vein. The nanoparticle concentration was determined using NanoDrop (Thermo Scientific).

### 3.7 Cell Isolation & Staining

Cells were isolated 72 hours after injection with LNPs unless otherwise noted. Mice were perfused with 20 mL of 1X PBS through the right atrium. Liver and lung tissues were finely minced, and then placed in a digestive enzyme solution with Collagenase Type I (Sigma Aldrich), Collagenase XI (Sigma Aldrich) and Hyaluronidase (Sigma Aldrich) at 37 ºC at 550 rpm for 45 minutes^4,5^. The spleen, bone marrow and thymus tissues were placed in 1X PBS solution. Cell suspension was filtered through 70μm mesh and red blood cells were lysed. Cells were stained to identify specific cell populations and sorted using the BD FacsFusion and BD Facs Aria IIIu cell sorters in the Georgia Institute of Technology Cellular Analysis Core. The antibody clones used were the following: anti‐CD31 (390, BioLegend), anti‐CD45.2 (104, BioLegend), anti‐CD68 (FA11, Biolegend), anti‐CD3 (17A2, Biolegend), anti‐CD19 (6D5, Biolegend), anti‐CD11b (M1/70, Biolegend), anit‐CD11c (N418, Biolegend), anti‐CD4 (GK1.5, Biolegend), anti‐CD8a (53‐6.7, Biolegend), anti‐CD34 (SA376A4, Biolegend), anti‐CD25 (3C7, Biolegend), anti‐CD326 (G8.8, Biolegend), and PE anti‐mCD47 (miap301, BioLegend). Representative flow gates are located in **Supplementary Figure 3**. PBS‐injected Ai14 mice were used to gate tdTomato populations for intravenous administration.

### 3.8 ddPCR

The QX200 Droplet Digital PCR System (BioRad) was used to analyze all ddPCR results. ddPCR samples were prepared with 10 μL of ddPCR with ddPCR Supermix for Probes (Bio‐Rad), 1 μL of primer and probe mix (solution of 10 μM target probe and 20 μM reverse/forward primers), 1 μL of template/TE buffer, and 8 μL of water. Once prepared, 20 μL of each reaction and 70 μL of Droplet Generation Oil for Probes (Bio‐Rad) were loaded into DG8 Cartridges and covered with DG8 Gaskets. Using the QX200 Droplet Generator, water−oil emulsion droplets were created. Cycle conditions for PCR was based on optimized conditions from BioRad. For each biological rep, three technical repetitions were completed. Unless stated otherwise, technical reps were averaged. Technical reps were only excluded if saturated was detected or there were inconsistent positive event amplitudes.

### 3.9 PCR Amplification

All samples were amplified and prepared for sequencing using a one‐step PCR protocol as previously described^3^. More specifically, 1 μL of primers (5 uM for Final Reverse/Forward, 0.5 uM for Base Forward) were added to 5 μL of Kapa HiFi 2X master mix, and 4 μL template DNA/water. When the PCR reaction did not produce clear bands, the primer concentrations, DNA template input, PCR temperature, and number of cycles were optimized for individual samples.

### 3.10 Deep Sequencing

Illumina deep sequencing was conducted in Georgia Tech’s Molecular Evolution core. Runs were performed on an Illumina Miniseq^TM^. Primers were designed based on Nextera XT adapter sequences.

### 3.11 Data Normalization and Analysis

Counts for each particle, per tissue, were normalized to the barcoded LNP mixture we injected into the mouse. This ‘input’ DNA provided the DNA counts and was used to normalize DNA counts from the cells and tissues.

Sequencing results were processed using a custom python‐based tool to extract raw barcode counts for each tissue. These raw counts were then normalized with an R script prior for further analysis. Statistical analysis was done using GraphPad Prism 8. Data is plotted as mean ± standard error mean unless otherwise stated.

### 3.12 Data Access

The data, analyses, and scripts used to generate all figures in the paper are available upon request from J.E.D. or dahlmanlab.org.

## 4 DISCUSSION

We believe these results are interesting for several reasons. First, mRNA delivery to non‐hepatocytes continues to be a significant roadblock to realizing the potential of RNA therapies. These data demonstrate that LNPs can deliver mRNA to liver microenvironment cells, without the use of traditional targeting ligands (e.g., antibodies, aptamers). Second, these data suggest that FIND in vivo assays can predict how individual LNPs deliver mRNA. More specifically, we characterized how 109 chemically distinct LNPs functionally delivered Cre mRNA to multiple cell types; using traditional 1‐by‐1 methods, this would require several hundred mice. Given that most LNP screens are performed in vitro, and given that in vitro delivery can poorly predict in vivo delivery, FIND may help accelerate the rate at which promising new LNP compositions are identified. Third, this report demonstrates that adamantyl‐based phospholipids can be incorporated into stable LNPs. Notably, all tested constrained phospholipid variants formed stable LNPs. Fourth, we found LNPs with unique compositions that deliver mRNA to non‐hepatic cell types. Specifically, we studied LNPs formulated with novel phospholipids containing a terminal quaternary ammonium headgroup, phosphodiester linkage, and an adamantyl group. We used FIND to identify an LNP, formulated phospholipid A‐11, that preferentially delivers to mRNA to Kupffer cells. These data support the claim that the addition of adamantyl groups into ionizable lipids can lead to interesting LNP tropism. Although the precise mechanisms governing constrained phospholipids require further study, we hypothesize the adamantyl group may influence the interactions between the LNP and the phospholipid bilayer on the surface of the cell, and potentially, subsequent endocytosis and endosomal release. Fifth, LNP1 delivery to Kupffer cells at a dose of 0.5 mg/kg was higher than previous MC3 delivery to Kupffer cells at a dose of 1.0 mg/kg using the same mRNA and same assay.^27^ Several additional “head‐to‐head” studies will be required to understand whether the relative efficiency is consistent. We therefore believe future work to explore the use of constrained phospholipids in nanoparticle formulations may be warranted to understand the mechanisms involved in the delivery of these unique LNPs.

It is also important to acknowledge several limitations to this work. First, it is possible that additional LNPs formulated with constrained phospholipids may not functionally deliver mRNA in vivo. Thus, many more experiments are required before constrained phospholipids are considered a validated new class of materials. Second, further in vivo optimization will be required to increase the potency of these compounds before they are clinically relevant; ideally, we would observe potent deliver at doses close to 0.01 mg/kg. Third, these LNPs did not require a targeting ligand to reach endothelial cells and Kupffer cells; the specific gene (or genes) that promote this tropism remain unknown at this time. Finally, these studies were performed in mice; it is feasible that these compounds do not behave similarly in non‐human primates, which are considered the gold standard pre‐clinical model for human beings. Despite these limitations, we believe these results demonstrate that a diverse and novel set of constrained phospholipids may be incorporated into LNPs with promising in vivo traits.

## CONFLICT OF INTEREST

James E. Dahlman is a co‐founder of Guide Therapeutics.

## Supporting information


**Appendix** **S1**: Supporting information.
