# Supplementary material for: Nanoparticles containing constrained phospholipids deliver mRNA to liver immune cells in vivo without targeting ligands
Source: Bioeng Transl Med. 2020 May 27;5(3):e10161. doi: 10.1002/btm2.10161 (PMC7974401; doi:10.1002/btm2.10161)
Supplement: Supplementary file 1 — Appendix S1: Supporting information. [file BTM2-5-e10161-s001.pdf]

Materials and Methods

General methods. Starting materials, reagents and solvents were purchased from commercial sources and used as received unless stated otherwise. Purification of reactions products was performed by column chromatography using silica gel (300-400 mesh) and eluting with hexane/ethyl acetate, DCM/MeOH. Thin layer chromatography (TLC) was carried out using precoated silica Gel GF plates and visualized using $KMnO_4$ stains. ^{1}H-NMR spectra were recorded at 400 or 500 MHz (Varian) using $CDCl_3$ with TMS. High-resolution mass spectra (HRMS) were recorded on LC/MS (Agilent Technologies 1260 Infinity II/6120 Quadrupole) and a time-of-flight mass spectrometer by ESI or matrix assisted laser desorption/ionization (MALDI).

Phospholipids Synthesis.

2-((3*r*,5*r*,7*r*)-Adamantan-1-yl)acetyl chloride (**2**)[1]

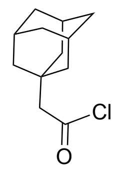

To a solution of 1-adamantaneacetic acid **1** (2.0 g, 10.3 mmol, 1 eq) and *N*,*N*-dimethylformamide (DMF) (0.1 mL, 0.1 mmol) in anhydrous DCM (30 mL), under nitrogen atmosphere, was added oxalyl chloride (2.6 mL, 30.9 mmol, 3 eq.) at 25 °C. The reaction mixture was refluxed at 60 ºC for 2 h and then concentrated under reduced pressure to provide crude product **2** as yellow oil (2.1 g, 95%), which was directly used in the next step without further purification.

(*R*)-3-(2-((3*R*,5*R*,7*R*)-Adamantan-1-yl)acetoxy)-2-hydroxypropyl(2-(trimethylammonio)ethyl) phosphate (**4**)

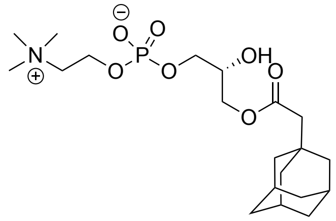

sn-Glycero-3-phosphocholine **3** (1.3 g, 5.0 mmol, 1 eq) and dibutyltin oxide (1.3 g, 5.5 mmol, 1.1 eq) were suspended in isopropanol (40 mL). The reaction mixture was refluxed for 2 h, then cooled down to 25 °C and treated with triethylamine (0.75 mL, 5.5 mmol, 1.1 eq). 2-((3*r*,5*r*,7*r*)-adamantan-1-yl)acetyl chloride **2** was dissolved in DCM (2 mL) and added to the reaction under nitrogen atmosphere. The reaction mixture was stirred for 1 h and then concentrated under

reduced pressure. The resulting residue was purified by flash chromatography (silica gel, gradient eluent: 10-20% of MeOH/DCM then 20% of MeOH/DCM containing with 1% ammonium hydroxide then 50% of MeOH/DCM containing with 10% ammonium hydroxide) to provide the desired product **4** (400 mg, 18 % yield) as colorless oil.

^{1}H NMR (400 MHz, $CDCl_3$/MeOD): δ 1.47 (br. s, 10H), 1.56 (br. s, 3H), 1.83 (s, 2H), 1.96 (t, *J* = 4.8 Hz, 2H), 3.08 (s, 3H), 3.48 (s, 2H), 3.71–3.74 (m, 1H), 3.83–3.84 (m, 1H), 3.96 (s, 3H), 4.13 (s, 2H).

^{13}C NMR (100 MHz, $CDCl_3$/MeOD): δ 28.3, 32.5, 36.4, 42.1, 53.9, 58.8, 64.4, 66.2, 66.8, 68.6, 171.9.

HRMS (ESI, m/z) calcd for $C_{20}H_{36}NNaO_7P$ $[M + Na]^+$: 456.2122, found 456.2118.

(*R*)-3-(2-((3*R*,5*R*,7*R*)-Adamantan-1-yl)acetoxy)-2-(decanoyloxy)propyl(2-(trimethylammonio) ethyl) phosphate (**A-9**)

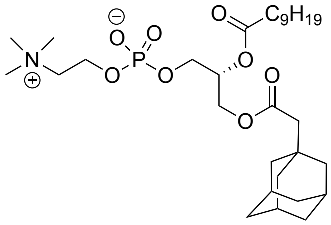

To a solution of product **4** (30 mg, 0.07 mmol, 1 eq), DMAP (3.7 mg, 0. 03 mmol, 0.4 eq), DIPEA (50.0 μL, 0.28 mmol, 4 eq) and decanoic acid (48 mg, 0.28 mmol, 4 eq) in anhydrous DCM (2 mL) was added *N*-(3-dimethylaminopropyl)-*N*'-ethylcarbodiimide hydrochloride (EDCI) (53 mg, 0.28 mmol, 4 eq) under nitrogen atmosphere. The reaction mixture was stirred at 25 °C for 16 h and then concentrated under reduced pressure. The resulting residue was purified by flash chromatography (silica gel, gradient eluent: 10-50% of MeOH/DCM then 50% of MeOH/DCM containing with 1-10% ammonium hydroxide) to provide the desired product **A-9** (38 mg, 92% yield) as colorless oil.

^{1}H NMR (500 MHz, $CDCl_3$): δ 0.87 (t, *J* = 7.5 Hz, 3H), 1.25 (br. s, 14H), 1.57 (s, 6H), 1.59–1.70 (m, 7H), 1.95 (s, 2H), 2.05 (s, 2H), 2.28 (td, *J* = 7.5, 3.5 Hz, 2H), 3.29 (s, 9H), 3.71 (br. s, 2H), 3.86–3.97 (m, 2H), 4.09–4.12 (m, 1H), 4.26 (br. s, 2H), 4.37 (dd, *J* = 12.0, 2.0 Hz, 1H), 5.17 (br. s, 1H).

^{13}C NMR (125 MHz, $CDCl_3$): δ 14.1, 22.7, 24.9, 28.5, 29.2, 29.3, 29.4, 29.5, 31.9, 32.7, 34.3, 36.7, 42.3, 48.8, 54.3, 59.4, 62.7, 63.5, 66.2, 70.5, 171.5, 173.2.

HRMS (ESI, m/z) calcd for $C_{30}H_{54}NNaO_8P$ $[M + Na]^+$: 610.3479, found 610.3468.

(*R*)-2-(2-((3*R*,5*R*,7*R*)-Adamantan-1-yl)acetoxy)-3-(undecanoyloxy)propyl(2-(trimethylammonio) ethyl) phosphate (**A-10**)

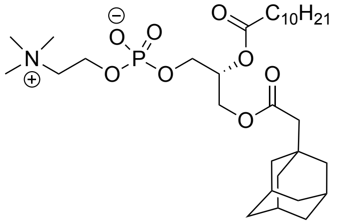

Product **A-10** was synthesized following the same procedure as product **A-9** using compound **4** (30 mg, 0.07 mmol, 1 eq), DMAP (3.7 mg, 0. 03 mmol, 0.4 eq), DIPEA (50.0 μL, 0.28 mmol, 4 eq) and undecanoic acid (52 mg, 0.28 mmol, 4 eq) in anhydrous CH_2Cl_2 (2 mL) was added EDCI (53 mg, 0.28 mmol, 4 eq) under nitrogen atmosphere and stirred overnight at 25 °C to provide **A-10** (34 mg, 78% yield) as a colorless oil.

^{1}H NMR (400 MHz, $CDCl_3$) δ 0.87 (t, *J* = 7.2 Hz, 3H), 1.25 (br. s, 16H), 1.57 (s, 6H), 1.57-1.70 (m, 7H), 1.94 (s, 2H), 2.05 (s, 2H), 2.28 (t, *J* = 7.2 Hz, 2H), 3.29 (s, 9H), 3.71 (br. s, 2H), 3.89-3.91 (m, 2H), 4.08–4.13 (m, 2H), 4.25 (br. s, 2H), 4.37 (dd, *J* = 12.0, 2.4 Hz, 1H), 5.17-5.18 (m, 1H).

^{13}C NMR (100 MHz, $CDCl_3$) δ 14.1, 22.7, 24.9, 28.6, 29.2, 29.3, 29.4, 29.6, 31.9, 32.7, 34.3, 36.7, 42.3, 48.8, 54.3, 59.4, 62.7, 63.4, 66.3, 70.5, 171.5, 173.2.

HRMS (ESI, m/z) calcd for $C_{31}H_{56}NNaO_8P$ $[M + Na]^+$: 624.3636, found 624.3624.

(*R*)-2-(2-((3*R*,5*R*,7*R*)-Adamantan-1-yl)acetoxy)-3-(dodecanoyloxy)propyl(2-(trimethylammonio) ethyl) phosphate (**A-11**)

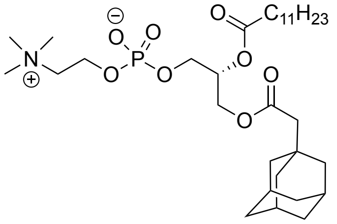

Product **A-11** was synthesized following the same procedure as product **A-9** using compound **4** (30 mg, 0.07 mmol, 1 eq), DMAP (3.7 mg, 0. 03 mmol, 0.4 eq), DIPEA (50.0 μL, 0.28 mmol, 4 eq) and lauric acid (56 mg, 0.28 mmol, 4 eq) in anhydrous CH_2Cl_2 (2 mL) was added EDCI (53 mg, 0.28 mmol, 4 eq) under nitrogen atmosphere and stirred overnight at 25 °C to provide **A-11** (38 mg, 85% yield) as a colorless oil.

^{1}H NMR (400 MHz, $CDCl_3$) δ 0.87 (t, *J* = 7.2 Hz, 3H), 1.25 (br. s, 18H), 1.56 (br. s, 6H), 1.59-1.70 (m, 7H), 1.94 (s, 2H), 2.04 (s, 2H), 2.28 (td, *J* = 7.2, 2.0 Hz, 2H), 3.29 (s, 9H), 3.71 (br. s, 2H),

3.87-3.95 (m, 2H), 4.08–4.13 (m, 1H), 4.26 (br. s, 2H), 4.37 (dd, *J* = 12.0, 2.4 Hz, 1H), 5.16-5.18 (m, 1H).

^{13}C NMR (100 MHz, $CDCl_3$) δ 14.1, 22.7, 24.9, 28.6, 29.3, 29.4, 29.5, 29.7, 29.8, 31.9, 32.7, 34.3, 36.7, 42.3, 48.7, 54.2, 59.4, 62.8, 63.6, 66.2, 70.5, 171.8, 173.5.

HRMS (ESI, m/z) calcd for $C_{32}H_{58}NNaO_8P$ $[M + Na]^+$: 638.3792, found 638.3780.

(*R*)-3-(2-((3*R*,5*R*,7*R*)-Adamantan-1-yl)acetoxy)-2-(tridecanoyloxy)propyl (2-(trimethylammonio) ethyl) phosphate (**A-12**)

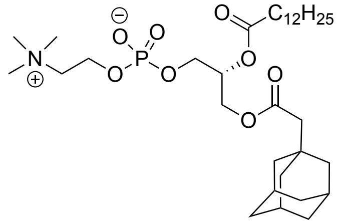

Product **A-12** was synthesized following the same procedure as product **A-9** using compound **4** (30 mg, 0.07 mmol, 1 eq), DMAP (3.7 mg, 0. 03 mmol, 0.4 eq), DIPEA (50.0 µL, 0.28 mmol, 4 eq) and tridecanoic acid (60 mg, 0.28 mmol, 4 eq) in anhydrous CH_2Cl_2 (2 mL) was added EDCI (53 mg, 0.28 mmol, 4 eq) under nitrogen atmosphere and stirred overnight at 25 °C to provide **A-12** (35 mg, 77% yield) as a colorless oil.

^{1}H NMR (400 MHz, $CDCl_3$) δ 0.87 (t, *J* = 6.8 Hz, 3H), 1.24 (br. s, 20H), 1.56 (br. s, 6H), 1.59-1.70 (m, 7H), 1.94 (br. s, 2H), 2.05 (s, 2H), 2.29 (td, *J* = 7.6 , 2.0 Hz, 2H), 3.29 (s, 9H), 3.71 (br. s, 2H), 3.89-3.91 (m, 2H), 4.08–4.13 (m, 1H), 4.25 (br. s, 2H), 4.37 (dd, *J* = 12.0, 2.4 Hz, 1H), 5.13-5.21 (m, 1H).

^{13}C NMR (100 MHz, $CDCl_3$) δ 14.1, 22.7, 24.9, 28.6, 29.2, 29.3, 29.4, 29.5, 29.6, 29.6, 29.7, 31.9, 32.7, 34.3, 36.7, 42.3, 48.8, 54.3, 59.4, 62.7, 63.4, 66.3, 70.5, 171.5, 173.2.

HRMS (ESI, m/z) calcd for $C_{33}H_{60}NNaO_8P$ $[M + Na]^+$: 652.3949, found 652.3939.

(*R*)-3-(2-((3*R*,5*R*,7*R*)-Adamantan-1-yl)acetoxy)-2-(tetradecanoyloxy)propyl(2(trimethylammonio) ethyl) phosphate (**A-13**)

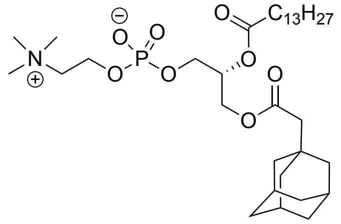

Product **A-13** was synthesized following the same procedure as product **A-9** using compound **4** (30 mg, 0.07 mmol, 4 eq), DMAP (3.7 mg, 0. 03 mmol, 0.4 eq), DIPEA (50.0 µL, 0.28 mmol, 4 eq) and myristic acid (64 mg, 0.28 mmol, 4 eq) in anhydrous CH_2Cl_2 (2 mL) under nitrogen atmosphere was added EDCI (53 mg, 0.28 mmol, 4 eq) under nitrogen atmosphere and stirred overnight at 25 °C to provide **A-13** (39 mg, 84% yield) as a colorless oil.

^{1}H NMR (400 MHz, $CDCl_3$) δ 0.87 (t, *J* = 7.2 Hz, 3H), 1.24-1.30 (m, 22H), 1.57 (br. s, 6H), 1.59-1.70 (m, 7H), 1.94 (br. s, 2H), 2.05 (s, 2H), 2.28 (td, *J* = 7.5, 2.4 Hz, 2H), 3.30 (s, 9H), 3.72-3.74 (m, 2H), 3.88-3.93 (m, 2H), 4.08–4.13 (m, 1H), 4.25-4.26 (m, 2H), 4.37 (dd, *J* = 12.0, 2.4 Hz, 1H), 5.17-5.18 (m, 1H).

^{13}C NMR (100 MHz, $CDCl_3$) δ 14.1, 22.7, 24.9, 28.6, 29.2, 29.3, 29.4, 29.6, 29.6, 29.7, 29.7, 31.9, 32.7, 34.3, 36.7, 42.3, 48.8, 54.3, 59.4, 62.7, 63.5, 66.2, 70.5, 171.5, 173.2.

HRMS (ESI, m/z) calcd for $C_{34}H_{63}NO_8P$ $[M + H]^+$: 644.4286, found 644.4278.

(*R*)-3-(2-((3*R*,5*R*,7*R*)-Adamantan-1-yl)acetoxy)-2-(pentadecanoyloxy)propyl(2(trimethyl--ammonio)ethyl) phosphate (**A-14**)

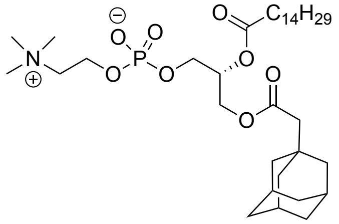

Product **A-14** was synthesized following the same procedure as product **A-9** using compound **4** (30 mg, 0.07 mmol, 1 eq), DMAP (3.7 mg, 0. 03 mmol, 0.4 eq), DIPEA (50.0 µL, 0.28 mmol, 4 eq) and pentadecanoic acid (68 mg, 0.28 mmol, 4 eq) in anhydrous CH_2Cl_2 (2 mL) was added EDCI (53 mg, 0.28 mmol, 4 eq) under nitrogen atmosphere and stirred overnight at 25 °C to provide **A-14** (39 mg, 82% yield) as a colorless oil.

^{1}H NMR (500 MHz, $CDCl_3$) δ 0.87 (t, *J* = 7.0 Hz, 3H), 1.24 (br. s, 24H), 1.57 (br. s, 6H), 1.59-1.70 (m, 7H), 1.94 (br. s, 2H), 2.04 (s, 2H), 2.26–2.30 (m, 2H), 3.29 (s, 9H), 3.72 (br. s, 2H), 3.89-3.93 (m, 2H), 4.08–4.12 (m, 1H), 4.25 (m, 2H), 4.37 (dd, *J* = 12.0, 2.0 Hz, 1H), 5.17-5.18 (m, 1H).

^{13}C NMR (125 MHz, $CDCl_3$) δ 14.1, 22.7, 24.9, 28.5, 29.2, 29.3, 29.4, 29.6, 29.6, 29.6, 29.7, 31.9, 32.7, 34.3, 36.7, 42.3, 48.7, 54.2, 59.4, 62.7, 63.5, 66.2, 70.5, 171.5, 173.2.

HRMS (ESI, m/z) calcd for $C_{35}H_{64}NNaO_8P$ $[M + Na]^+$: 680.4262, found 680.4253.

(*R*)-3-(2-((3*R*,5*R*,7*R*)-Adamantan-1-yl)acetoxy)-2-(palmitoyloxy)propyl (2-(trimethylammonio) ethyl) phosphate (**A-15**)

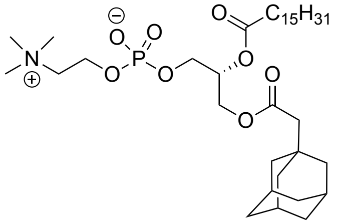

Product **A-15** was synthesized following the same procedure as product **A-9** using compound **4** (30 mg, 0.07 mmol, 1 eq), DMAP (3.7 mg, 0. 03 mmol, 0.4 eq), DIPEA (50.0 µL, 0.28 mmol, 4 eq) and palmitic acid (72 mg, 0.28 mmol, 4 eq) in anhydrous CH_2Cl_2 (2 mL) was added EDCI (53 mg, 0.28 mmol, 4 eq) under nitrogen atmosphere and stirred overnight at 25 °C to provide **A-15** (43 mg, 89% yield) as a colorless oil.

^{1}H NMR (500 MHz, $CDCl_3$) δ 0.87 (t, *J* = 7.0 Hz, 3H), 1.24 (br. s, 26H), 1.57 (s, 6H), 1.67-1.70 (m, 7H), 1.94 (s, 2H), 2.05 (s, 2H), 2.26–2.30 (m, 2H), 3.28 (s, 9H), 3.70 (s, 2H), 3.89–3.92 (m, 2H), 4.08–4.12 (m, 1H), 4.25–4.38 (m, 2H), 5.17 (br. s, 1H).

^{13}C NMR (125 MHz, $CDCl_3$) δ 14.1, 22.7, 24.9, 28.5, 29.2, 29.3, 29.4, 29.6 (two carbons overlapped each other), 29.7 (two carbons overlapped each other), 31.9, 32.7, 34.3, 36.7, 42.3, 48.8, 54.2, 59.4, 62.7, 63.5, 66.2, 70.5, 171.5, 173.2.

HRMS (ESI, m/z) calcd for $C_{36}H_{66}NNaO_8P$ $[M + Na]^+$: 694.4418, found 694.4417.

(R)-3-(2-((3R,5R,7R)-Adamantan-1-yl)acetoxy)-2-(heptadecanoyloxy)propyl(2-(trimethyl- -ammonio)ethyl) phosphate (**A-16**)

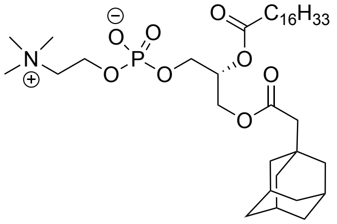

Product **A-16** was synthesized following the same procedure as product **A-9** using compound **4** (30 mg, 0.07 mmol, 1 eq), DMAP (3.7 mg, 0. 03 mmol, 0.4 eq), DIPEA (50.0 µL, 0.28 mmol, 4 eq) and heptadecanoic acid (76 mg, 0.28 mmol, 4 eq) in anhydrous CH_2Cl_2 (2 mL) was added EDCI (53 mg, 0.28 mmol, 4 eq) under nitrogen atmosphere and stirred overnight at 25 °C to provide **A-16** (38 mg, 77% yield) as a colorless oil.

^{1}H NMR (400 MHz, $CDCl_3$) δ 0.87 (t, *J* = 7.0 Hz, 3H), 1.24 (br. s, 28H), 1.57 (s, 6H), 1.59-1.67 (m, 7H), 1.94 (s, 2H), 2.05 (s, 2H), 2.28–2.31 (m, 2H), 3.30 (s, 9H), 3.73 (s, 2H), 3.92 (br. s, 2H), 4.10–4.11 (m, 1H), 4.35–4.38 (m, 2H), 5.17 (br. s, 1H).

^{13}C NMR (100 MHz, $CDCl_3$) δ 14.1, 22.7, 24.9, 28.6, 29.2, 29.3, 29.4, 29.6 (two carbons overlapped each other), 29.7 (three carbons overlapped each other), 31.9, 32.7, 34.3, 36.7, 42.3, 48.8, 54.3, 59.5, 62.7, 63.5, 66.2, 70.5, 171.5, 173.2.

HRMS (ESI, m/z) calcd for $C_{37}H_{68}NO_8P$ $[M + Na]^+$: 708.4575, found 708.4565.

(R)-3-(2-((3R,5R,7R)-Adamantan-1-yl)acetoxy)-2-(stearoyloxy)propyl (2-(trimethylammonio) ethyl) phosphate (**A-17**)

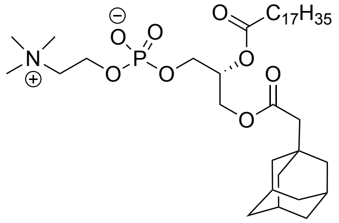

Product **A-17** was synthesized following the same procedure as product **A-9** using compound **4** (30 mg, 0.07 mmol, 1 eq), DMAP (3.7 mg, 0. 03 mmol, 0.4 eq), DIPEA (50.0 µL, 0.28 mmol, 4 eq) and stearic acid (79 mg, 0.28 mmol, 4 eq) in anhydrous CH_2Cl_2 (2 mL) was added EDCI (53 mg, 0.28 mmol, 4 eq) under nitrogen atmosphere and stirred overnight at 25 °C to provide **A-17** (39 mg, 77% yield) as a colorless oil.

^{1}H NMR (400 MHz, $CDCl_3$) δ 0.87 (t, *J* = 7.2 Hz, 3H), 1.24 (br. s, 30H), 1.57 (s, 6H), 1.59-1.70 (m, 7H), 1.95 (s, 2H), 2.05 (s, 2H), 2.26–2.31 (m, 2H), 3.30 (s, 9H), 3.73 (s, 2H), 3.91–3.92 (m, 2H), 4.10–4.27 (m, 1H), 4.35–4.39 (m, 2H), 5.17–5.18 (m, 1H).

^{13}C NMR (100 MHz, $CDCl_3$) δ 14.1, 22.7, 25.0, 28.6, 29.3, 29.4 (two carbons overlapped each other), 29.6, 29.7 (two carbons overlapped each other), 29.8, 31.9, 32.7, 34.4, 36.7, 42.3, 48.8, 54.3, 59.4, 62.7, 63.5, 66.3, 70.5, 171.5, 173.2.

HRMS (ESI, m/z) calcd for $C_{38}H_{70}NNaO_8P$ $[M + Na]^+$: 722.4731, found 722.4723.

(R)-3-(2-((3R,5R,7R)-Adamantan-1-yl)acetoxy)-2-(((9Z,12Z)-octadeca-9,12-dienoyl)oxy)propyl (2-(trimethylammonio)ethyl) phosphate (**A-17-2Z**)

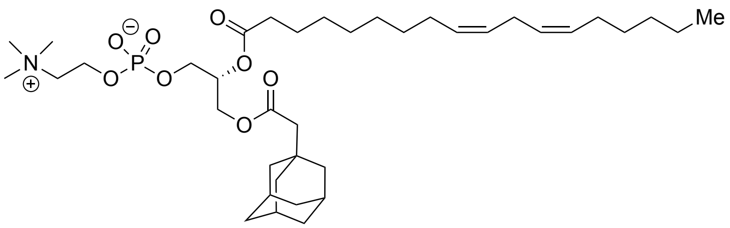

Product **A-17-2Z** was synthesized following the same procedure as product **A-9** using compound **4** (30 mg, 0.07 mmol, 1 eq), DMAP (3.7 mg, 0. 03 mmol, 0.4 eq), DIPEA (50.0 µL, 0.28 mmol, 4 eq) and linoleic acid (79 mg, 0.28 mmol, 4 eq) in anhydrous CH_2Cl_2 (2 mL) was added EDCI (53 mg, 0.28 mmol, 4 eq.) under nitrogen atmosphere and stirred overnight at 25 °C to provide **A-17-2Z** (41 mg, 82% yield) as a colorless oil.

^{1}H NMR (400 MHz, $CDCl_3$) δ 0.87 (t, *J* = 7.2 Hz, 3H), 1.28 (br. s, 16H), 1.57 (s, 6H), 1.59-1.70 (m, 7H), 1.94 (s, 2H), 1.99-2.04 (m, 7H), 2.26-2.30 (m, 2H), 2.75 (t, *J* = 6.8 Hz, 1H), 3.29 (s, 9H), 3.70 (br. s, 2H), 3.84-3.97 (m, 2H), 4.08–4.13 (m, 1H), 4.25 (s, 2H), 4.36–4.39 (m, 1H), 5.13-5.21 (m, 1H), 5.26-5.43 (m, 3H).

^{13}C NMR (100 MHz, $CDCl_3$) δ 14.0, 22.5, 24.9, 25.6, 27.2, 28.6, 29.2, 29.3, 29.5, 29.7, 29.8, 31.5, 31.9, 32.7, 34.3, 36.7, 42.3, 48.8, 54.3, 59.4, 62.7, 63.5, 66.2, 70.5, 127.8, 128.0, 130.0, 130.2, 171.5, 173.1.

HRMS (ESI, m/z) calcd for $C_{38}H_{66}NNaO_8P$ $[M + Na]^+$: 718.4418, found 718.4399.

Nanoparticle Formulation. Nanoparticles were formulated with a NanoAssemblr Spark Formulation Device (Precision Nanosystems). Briefly, nucleic acids (DNA barcodes, mRNA, and sgRNA) were diluted in 10mM citrate buffer (Teknova). Lipid-amine compounds, alkyl-tailed PEG, cholesterol, and helper lipids were diluted in 100% ethanol. Both phases were loaded into the NanoAssemblr Spark Cartridge. An optimized protocol, designed by Precision Nanosystems for the NanoAssemblr Spark, was followed. For nanoparticle screens, Cre mRNA and DNA barcodes were mixed at a 10:1 mass ratio. All PEGs and cholesterol were purchased from Avanti Lipids.

DNA Barcoding. Each LNP was formulated to carry a unique DNA barcode. LNP1 carried DNA barcode 1, while the chemically distinct LNPN carried DNA barcode N. DNA barcodes were designed rationally with several characteristics, as we previously described[2,3]. Single stranded DNA barcodes were purchased from Integrated DNA Technologies. Each barcode was

distinguished using a unique 8 nucleotide sequence. An 8-nucleotide sequence can generate over 4^8 (65,536) distinct barcodes. We used distinct 8 nucleotide sequences designed by to prevent sequence bleaching and reading errors on the Illumina MiniSeq™ sequencing machine.

Nanoparticle Characterization. LNP hydrodynamic diameter was measured using high throughput dynamic light scattering (DLS) (DynaPro Plate Reader II, Wyatt). LNPs were diluted in sterile 1X PBS and analyzed. To avoid using unstable LNPs, and to enable sterile purification using a 0.22 µm filter, LNPs were included only if they met 3 criteria: diameter >20 nm, diameter <200 nm, and correlation function with 1 inflection point. Particles that met these criteria were dialyzed with 1X PBS.

Animal Experiments. All animal experiments were performed in accordance with the Georgia Institute of Technology's IACUC. All animals were bread in the Georgia Institute of Technology Animal Facility. C57BL/6J (#000664) were purchased from The Jackson Laboratory. LSL-Tomato/Ai14 (#007914) were purchased from The Jackson Laboratory for breeding proposes. In all experiments, we used N=3-5 mice / group. Mice were injected intravenously via the lateral tail vein. The nanoparticle concentration was determined using NanoDrop (Thermo Scientific).

Cell Isolation & Staining. Cells were isolated 72 hours after injection with LNPs unless otherwise noted. Mice were perfused with 20 mL of 1X PBS through the right atrium. Liver and lung tissues were finely minced, and then placed in a digestive enzyme solution with Collagenase Type I (Sigma Aldrich), Collagenase XI (Sigma Aldrich) and Hyaluronidase (Sigma Aldrich) at 37 ºC at 550 rpm for 45 minutes[4,5]. The spleen, bone marrow and thymus tissues were placed in 1X PBS solution. Cell suspension was filtered through 70µm mesh and red blood cells were lysed. Cells were stained to identify specific cell populations and sorted using the BD FacsFusion and BD Facs Aria IIIu cell sorters in the Georgia Institute of Technology Cellular Analysis Core. The antibody clones used were the following: anti-CD31 (390, BioLegend), anti-CD45.2 (104, BioLegend), anti-CD68 (FA11, Biolegend), anti-CD3 (17A2, Biolegend), anti-CD19 (6D5, Biolegend), anti-CD11b (M1/70, Biolegend), anit-CD11c (N418, Biolegend), anti-CD4 (GK1.5, Biolegend), anti-CD8a (53-6.7, Biolegend), anti-CD34 (SA376A4, Biolegend), anti-CD25 (3C7, Biolegend), anti-CD326 (G8.8, Biolegend), and PE anti-mCD47 (miap301, BioLegend). Representative flow gates are located in **Supplementary Figure 3**. PBS-injected Ai14 mice were used to gate tdTomato populations for intravenous administration.

ddPCR. The QX200 Droplet Digital PCR System (BioRad) was used to analyze all ddPCR results. ddPCR samples were prepared with 10 μL of ddPCR with ddPCR Supermix for Probes (Bio-Rad), 1 μL of primer and probe mix (solution of 10 μM target probe and 20 μM reverse/forward primers), 1 μL of template/TE buffer, and 8 μL of water. Once prepared, 20 μL of each reaction and 70 μL of Droplet Generation Oil for Probes (Bio-Rad) were loaded into DG8 Cartridges and covered with DG8 Gaskets. Using the QX200 Droplet Generator, water−oil emulsion droplets were created. Cycle conditions for PCR was based on optimized conditions from BioRad. For each biological rep, three technical repetitions were completed. Unless stated otherwise, technical reps were averaged. Technical reps were only excluded if saturated was detected or there were inconsistent positive event amplitudes.

PCR Amplification. All samples were amplified and prepared for sequencing using a one-step PCR protocol as previously described[3]. More specifically, 1 μL of primers (5 uM for Final Reverse/Forward, 0.5 uM for Base Forward) were added to 5 μL of Kapa HiFi 2X master mix, and 4 μL template DNA/water. When the PCR reaction did not produce clear bands, the primer concentrations, DNA template input, PCR temperature, and number of cycles were optimized for individual samples.

Deep Sequencing. Illumina deep sequencing was conducted in Georgia Tech's Molecular Evolution core. Runs were performed on an Illumina Miniseq™. Primers were designed based on Nextera XT adapter sequences.

Data Normalization and Analysis. Counts for each particle, per tissue, were normalized to the barcoded LNP mixture we injected into the mouse. This 'input' DNA provided the DNA counts and was used to normalize DNA counts from the cells and tissues.

Sequencing results were processed using a custom python-based tool to extract raw barcode counts for each tissue. These raw counts were then normalized with an R script prior for further analysis. Statistical analysis was done using GraphPad Prism 8. Data is plotted as mean ± standard error mean unless otherwise stated.

Data Access. The data, analyses, and scripts used to generate all figures in the paper are available upon request from J.E.D. or dahlmanlab.org.

4

^{1}H-NMR (400 MHz, $CDCl_3$/MeOD)

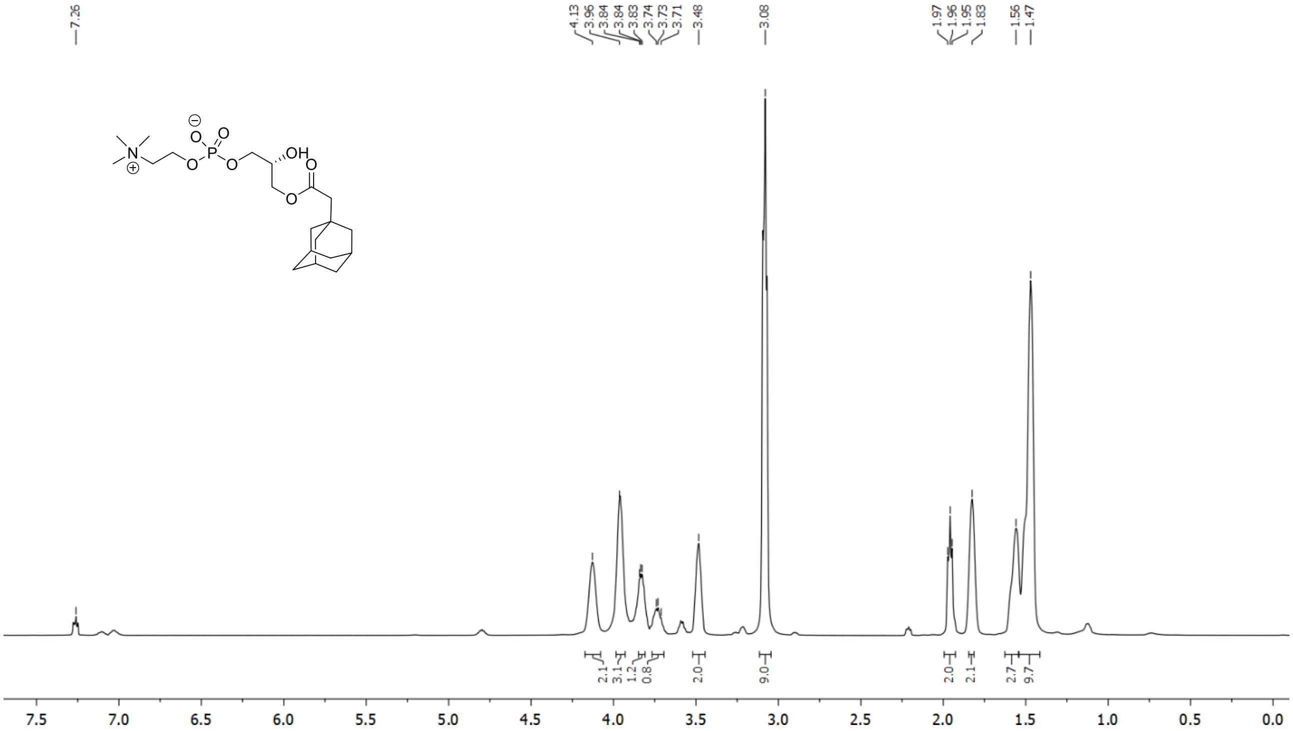

4

^{13}C-NMR (400 MHz, $CDCl_3$/MeOD)

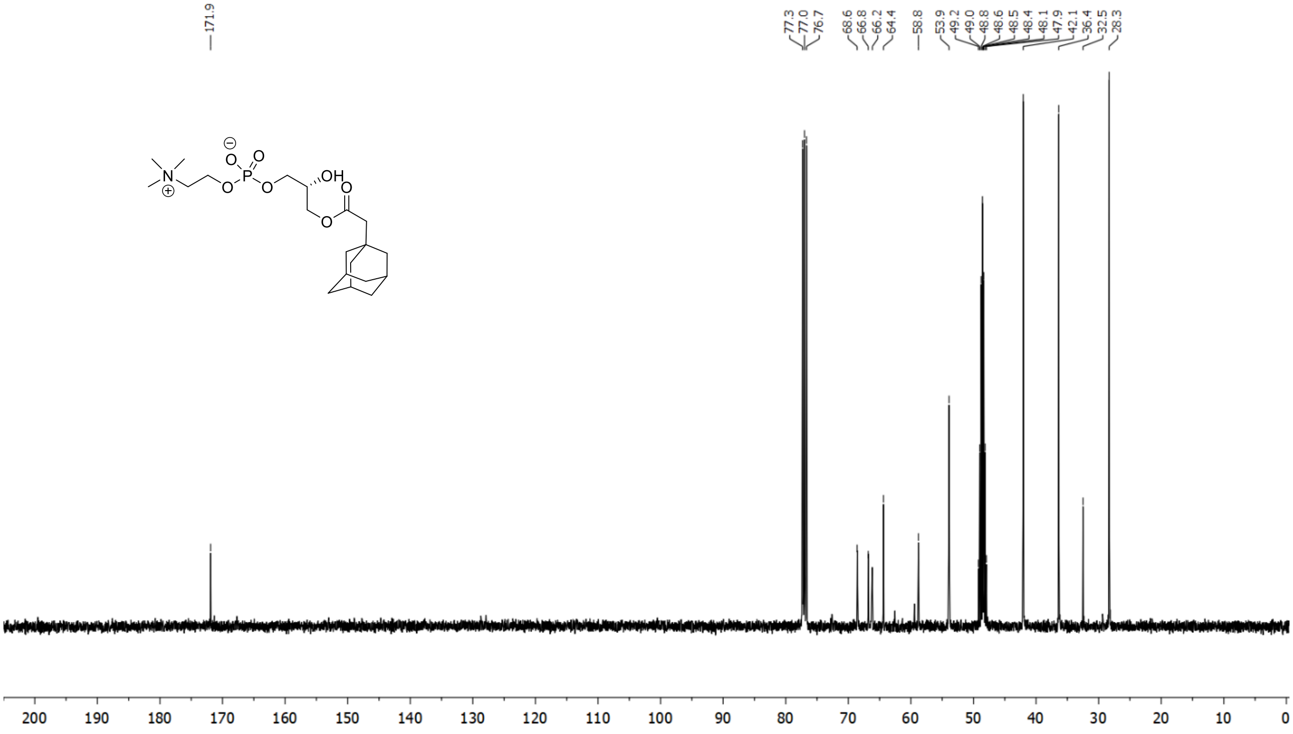

A-9
^{1}H-NMR (500 MHz, $CDCl_3$)

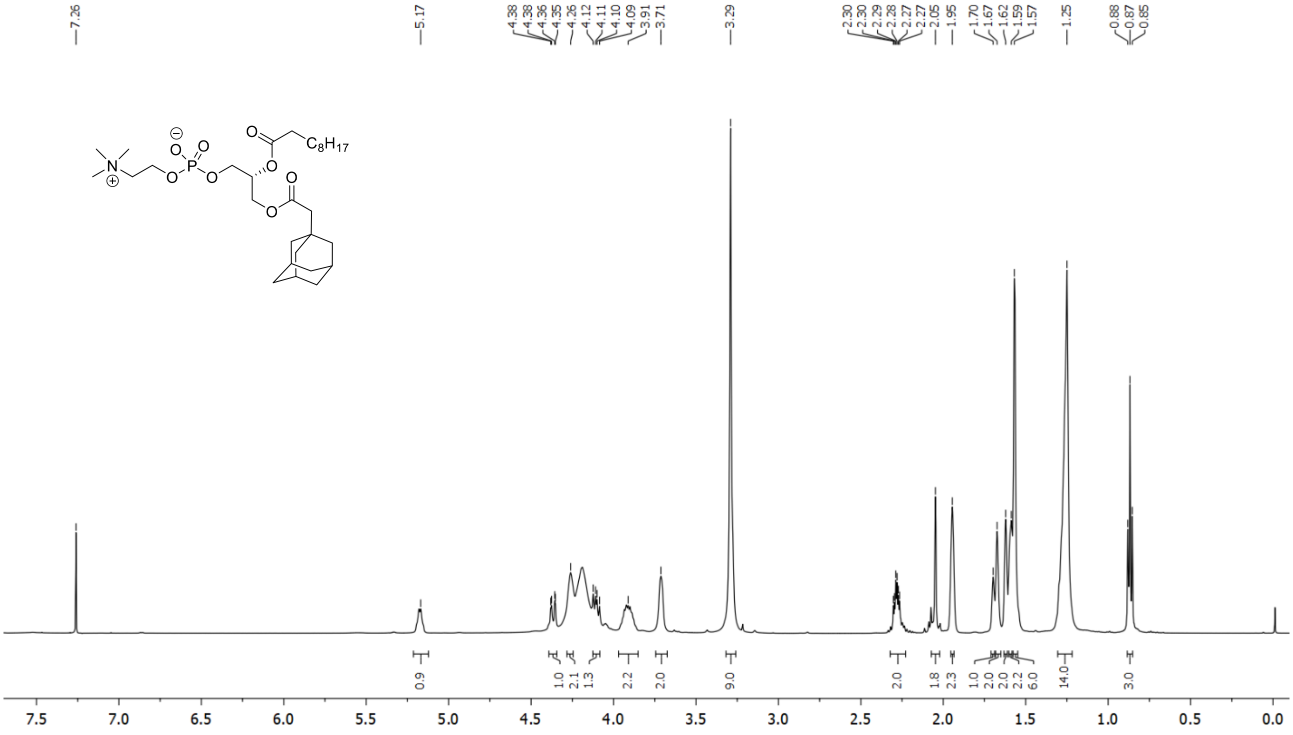

A-9
^{13}C-NMR (125 MHz, $CDCl_3$)

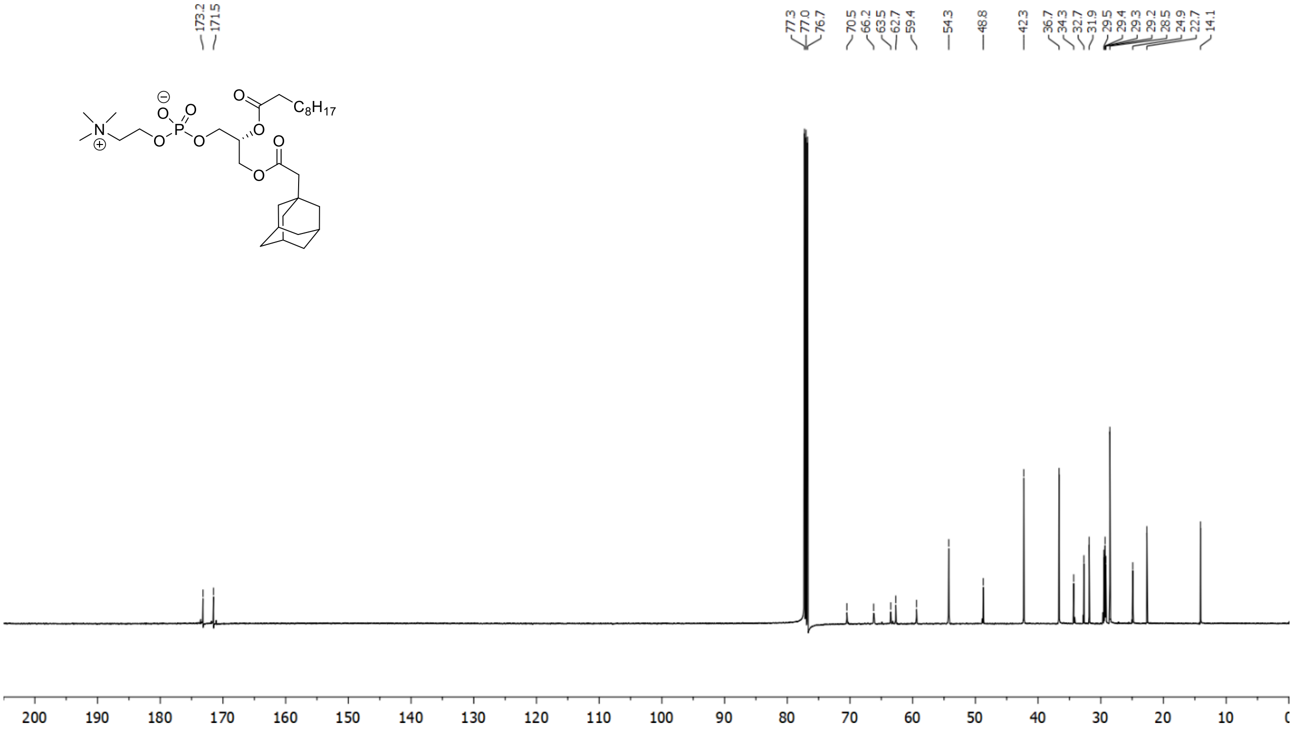

A-10

^{1}H-NMR (400 MHz, $CDCl_3$)

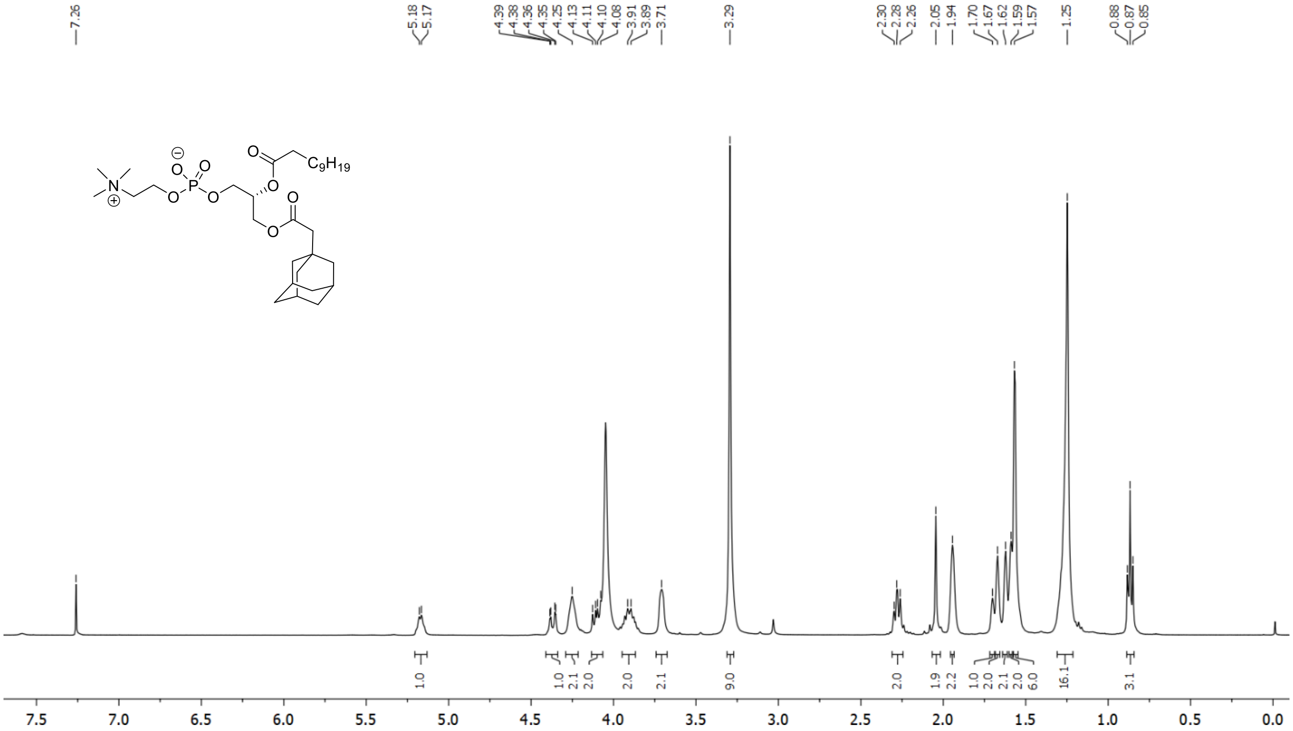

A-10

^{13}C-NMR (100 MHz, $CDCl_3$)

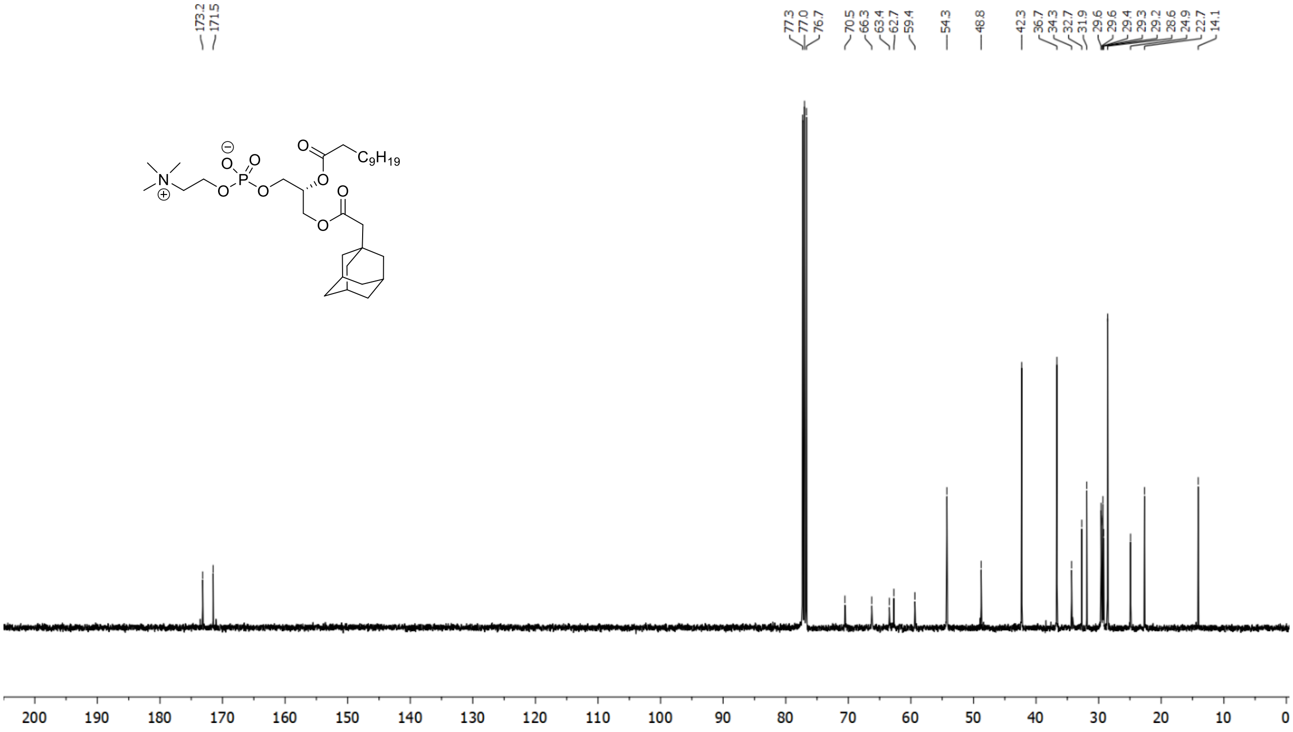

A-11

^{1}H-NMR (400 MHz, $CDCl_3$)

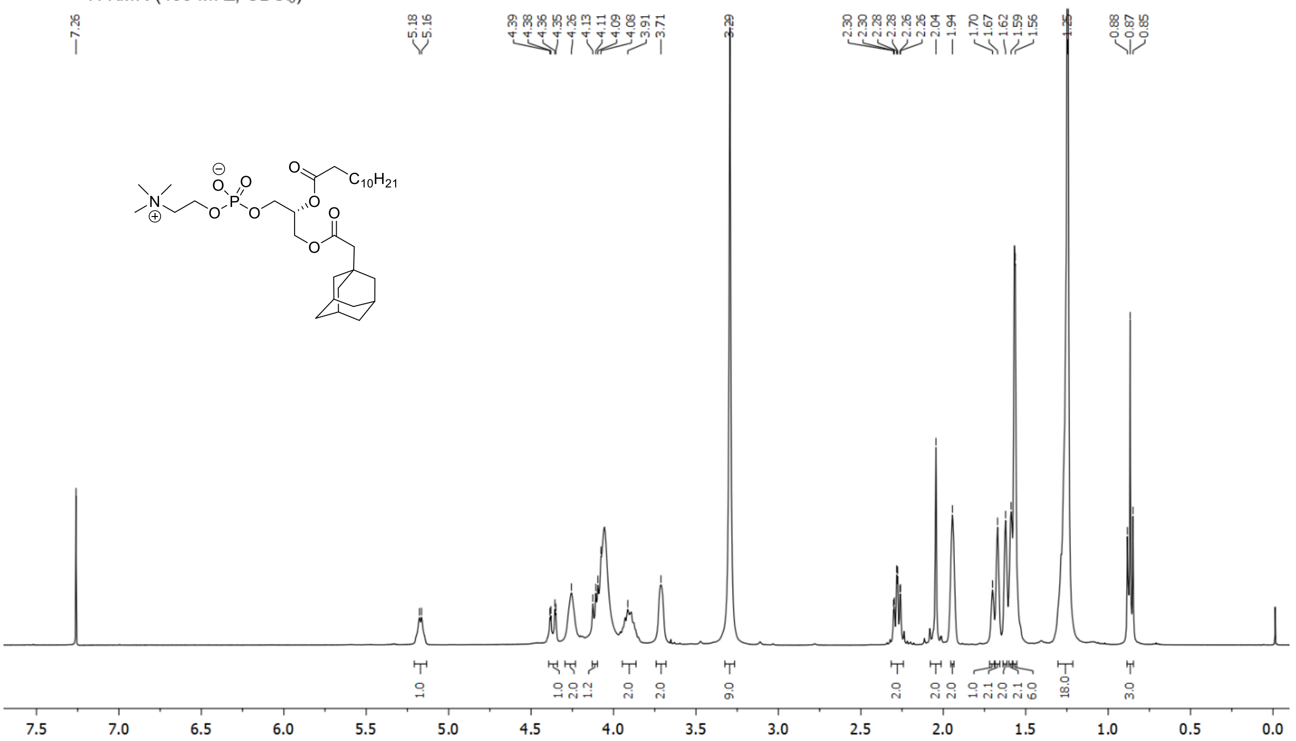

A-11

^{13}C-NMR (100 MHz, $CDCl_3$)

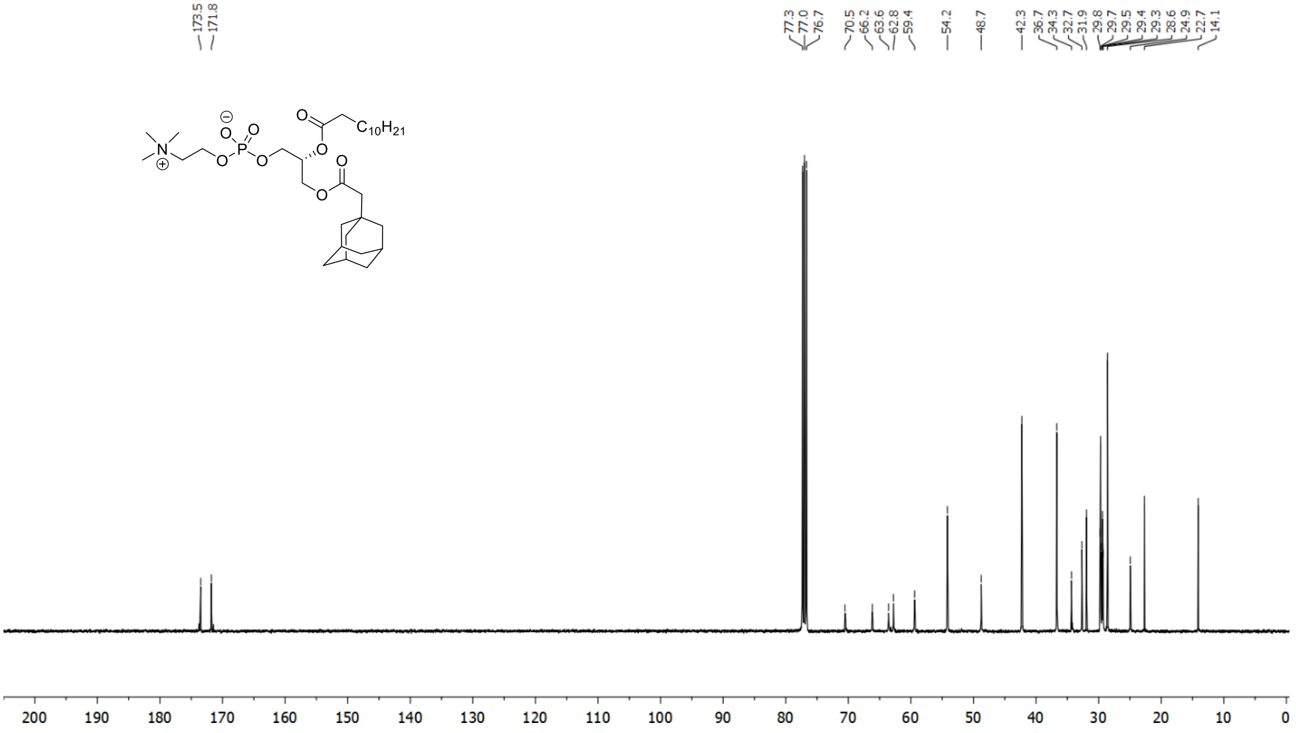

A-12

^{1}H-NMR (400 MHz, $CDCl_3$)

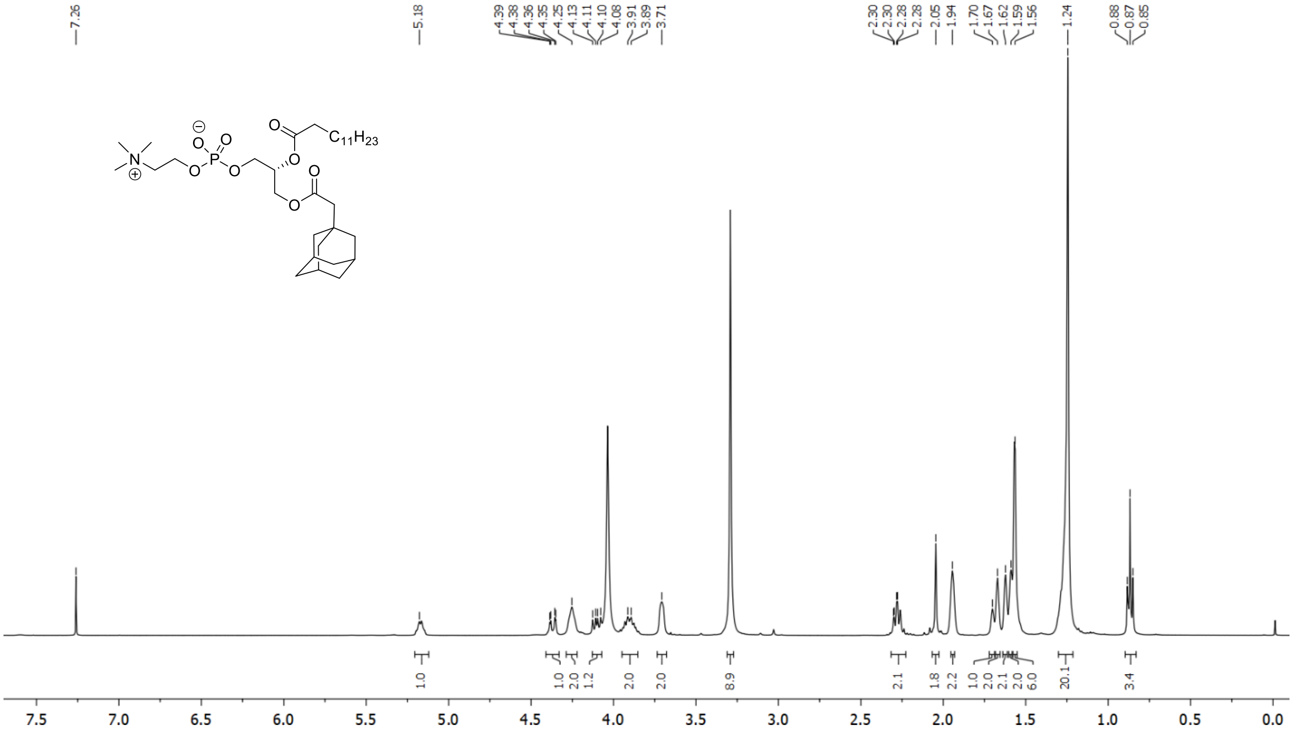

A-12

^{13}C-NMR (100 MHz, $CDCl_3$)

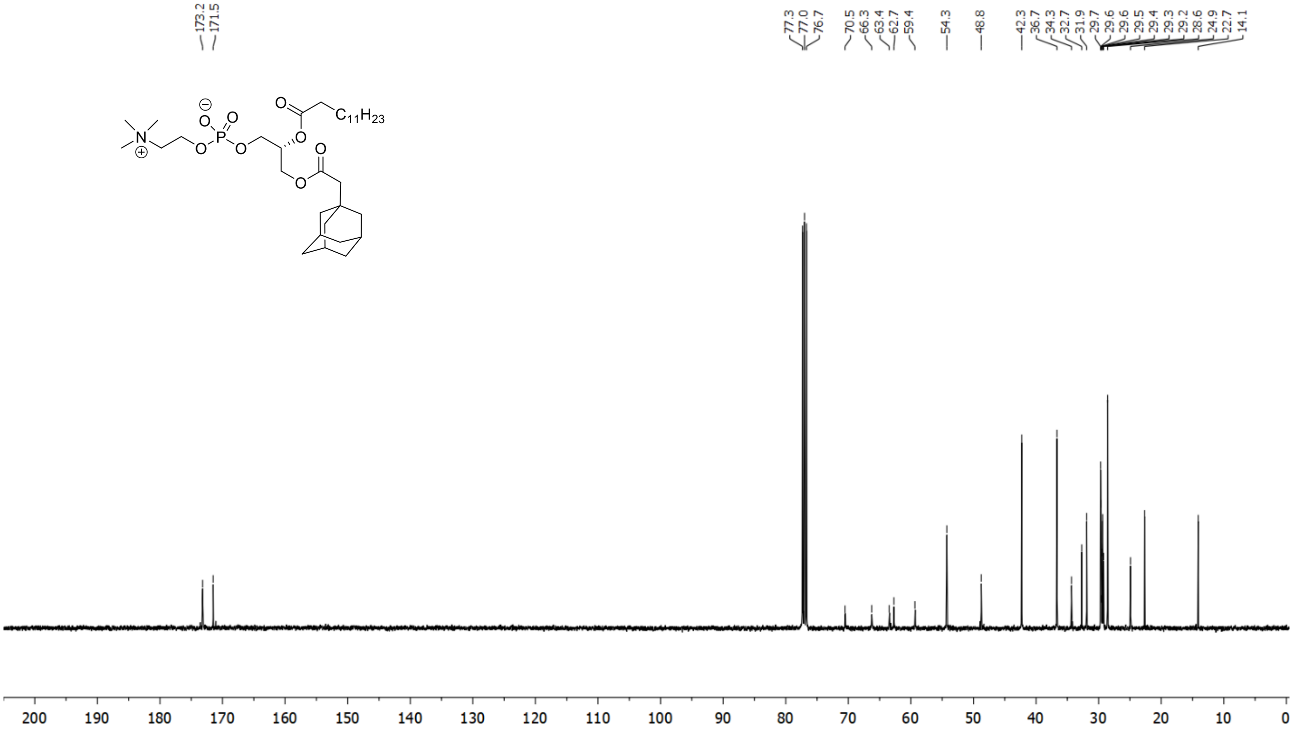

A-13
^{1}H-NMR (400 MHz, $CDCl_3$)

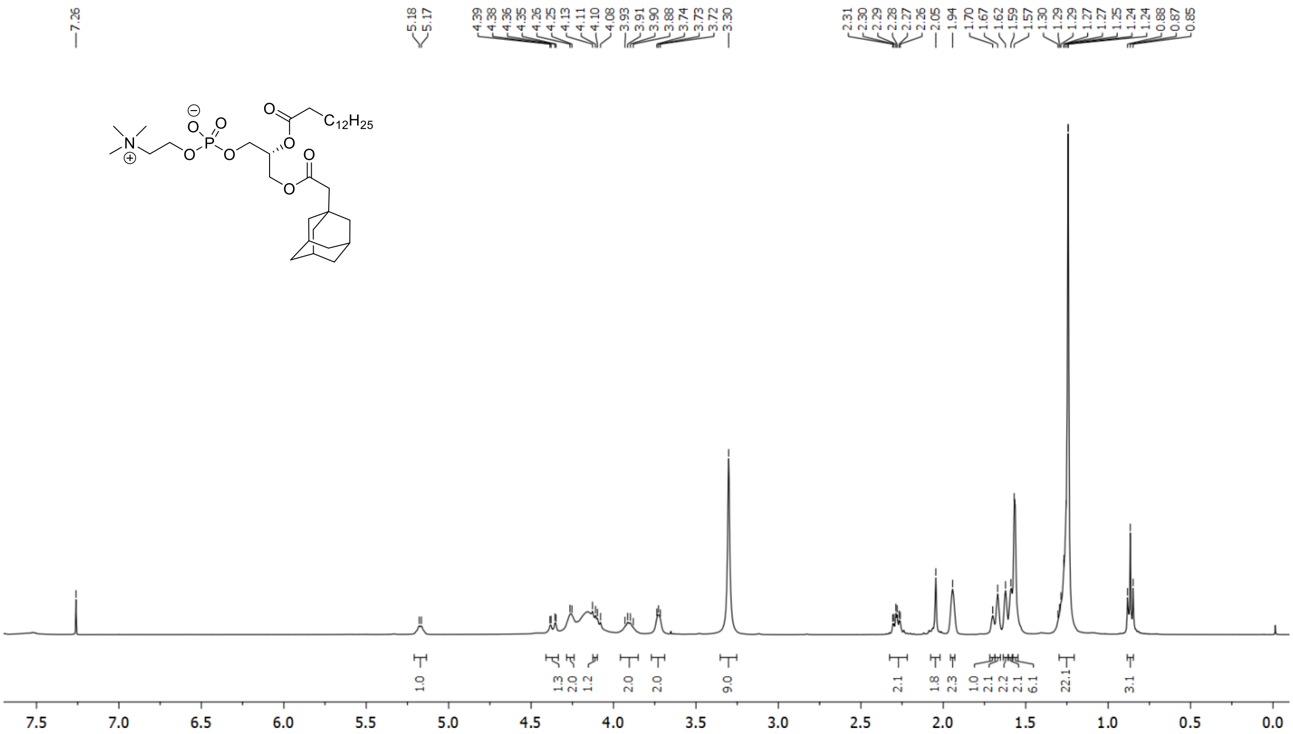

A-13
^{13}C-NMR (100 MHz, $CDCl_3$)

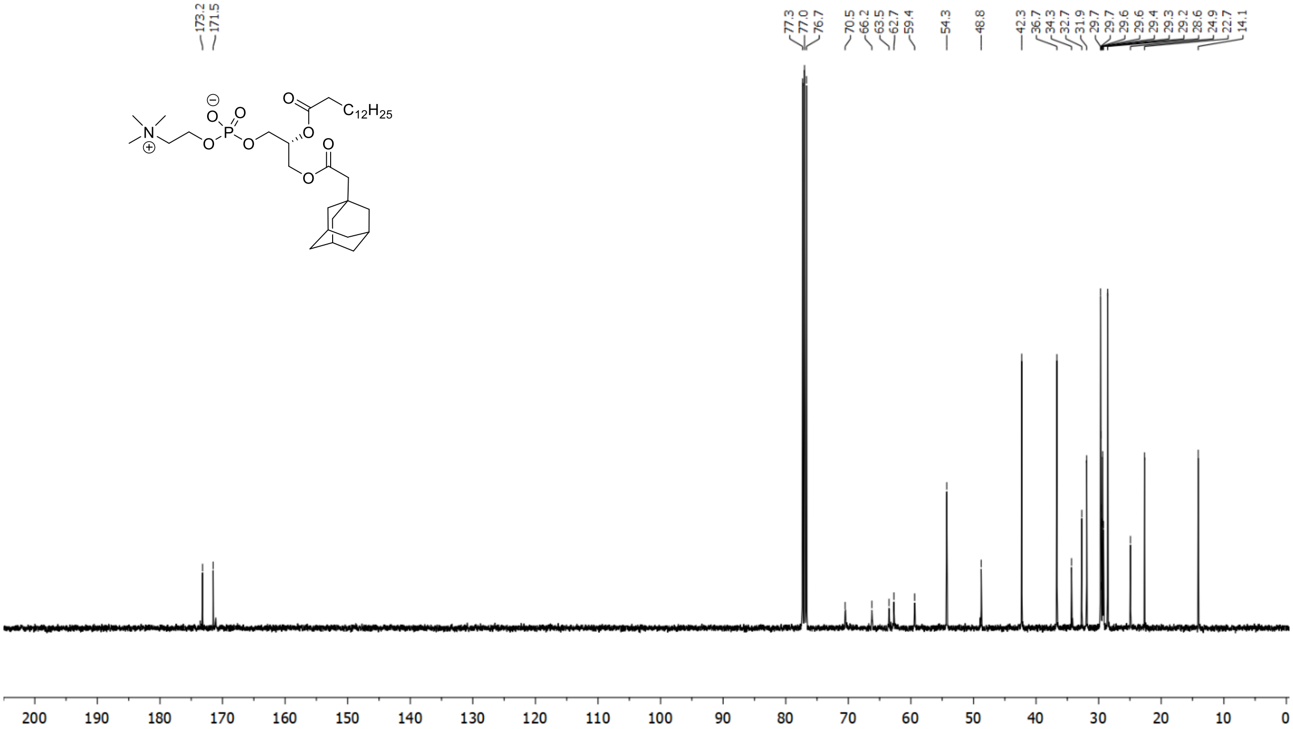

A-14

^{1}H-NMR (500 MHz, CDCl$_3$)

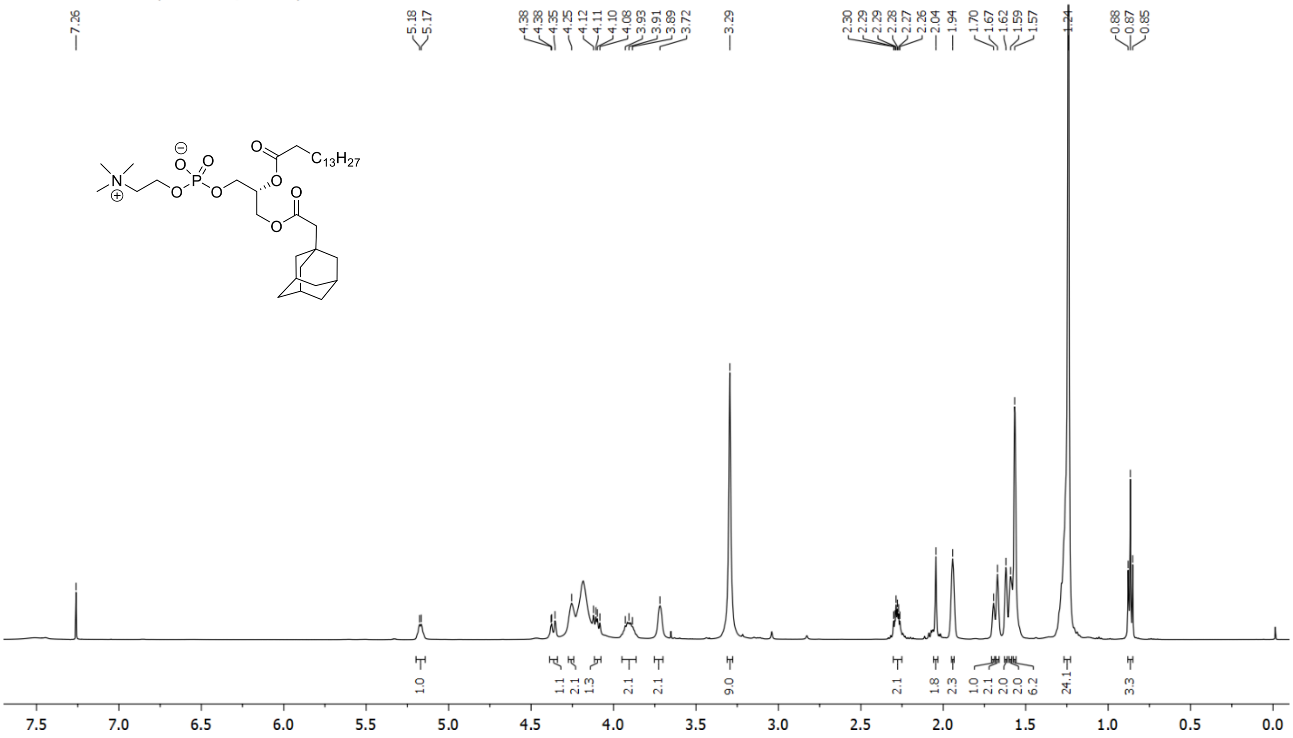

A-14

^{13}C-NMR (125 MHz, CDCl$_3$)

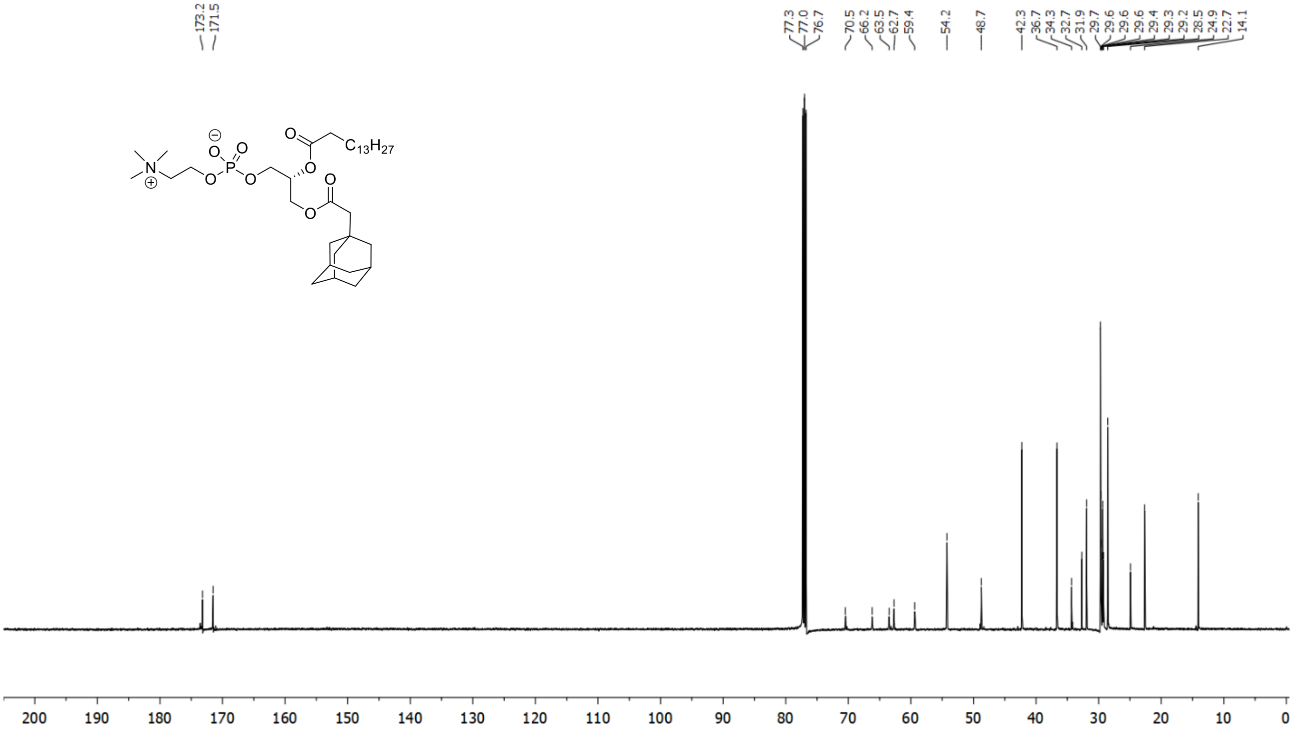

A-15

^{1}H-NMR (500 MHz, $CDCl_3$)

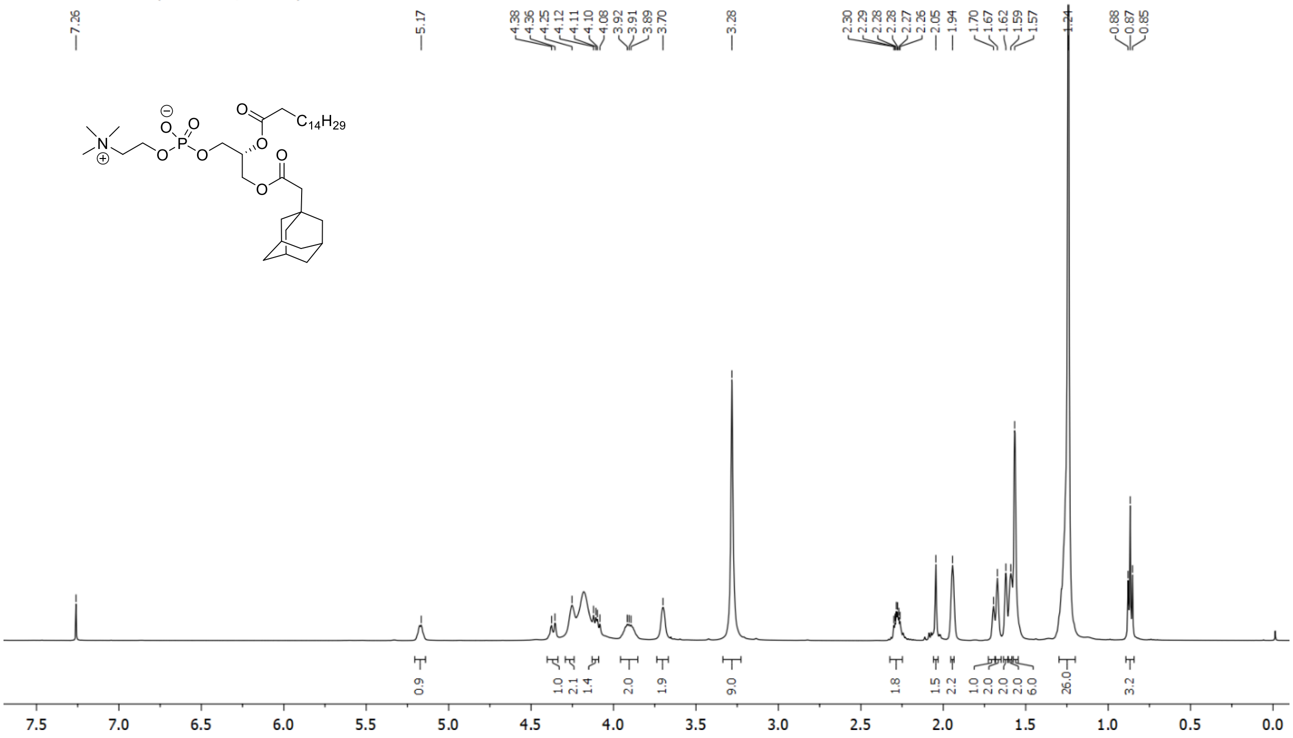

A-15

^{13}C-NMR (125 MHz, $CDCl_3$)

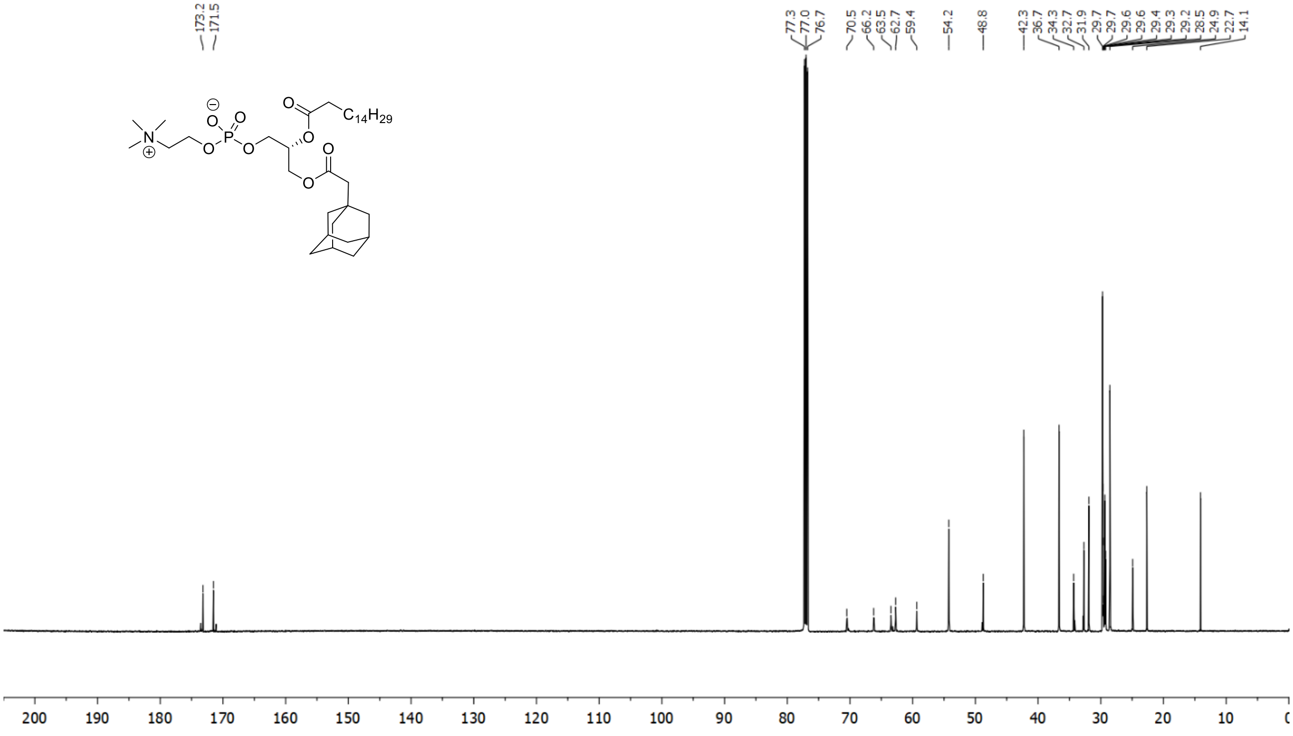

A-16

^{1}H-NMR (400 MHz, $CDCl_3$)

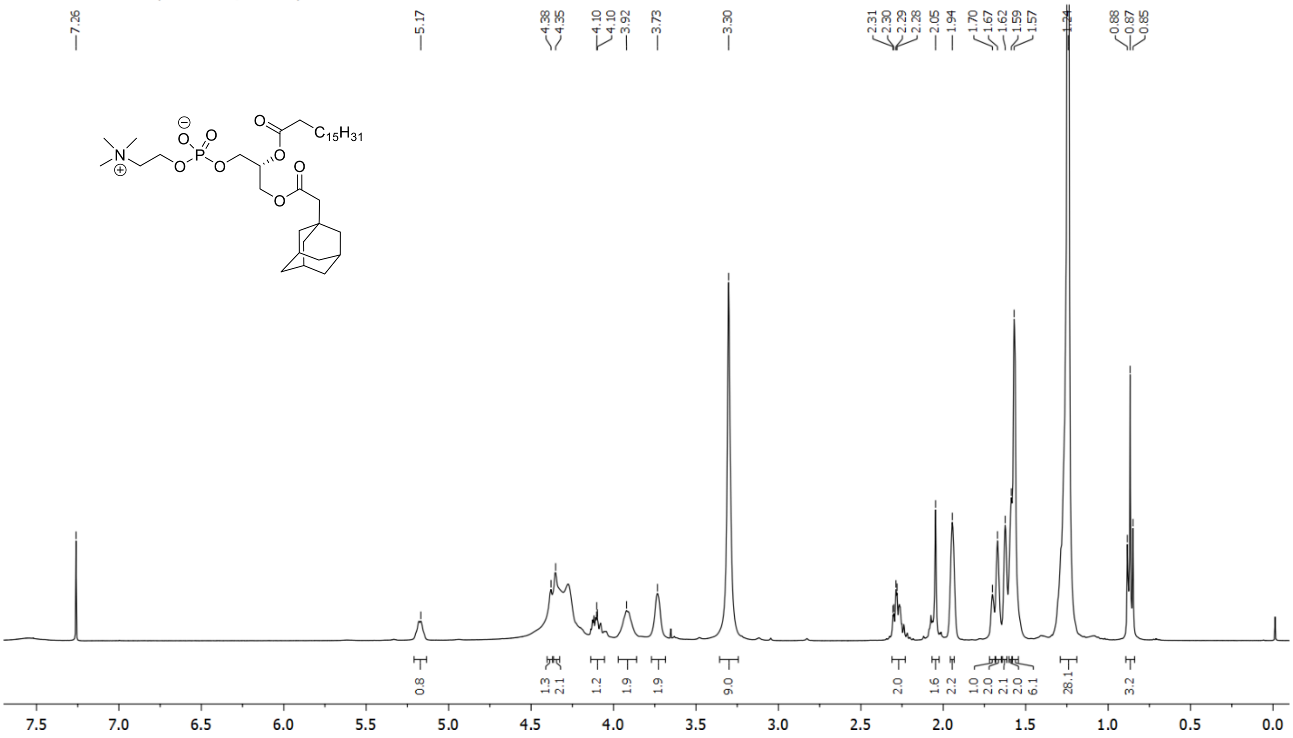

A-16

^{13}C-NMR (100 MHz, $CDCl_3$)

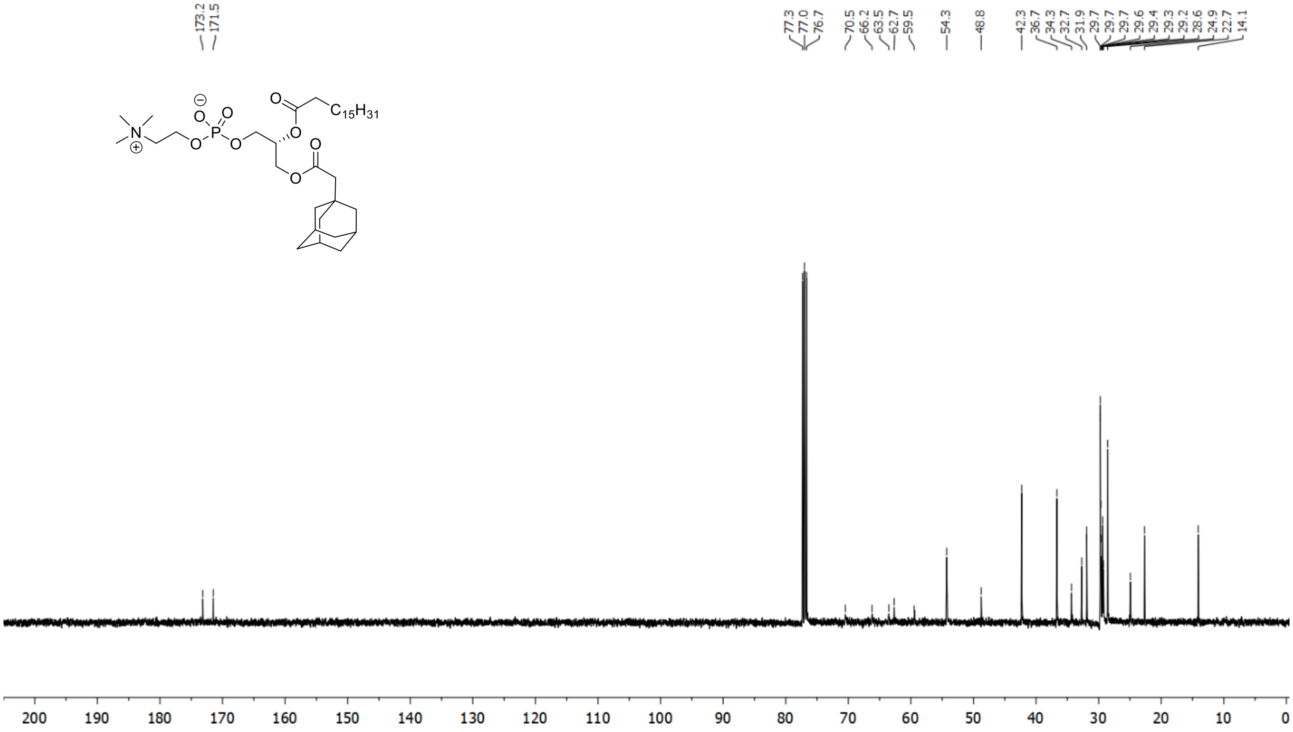

A-17

^{1}H-NMR (400 MHz, $CDCl_3$)

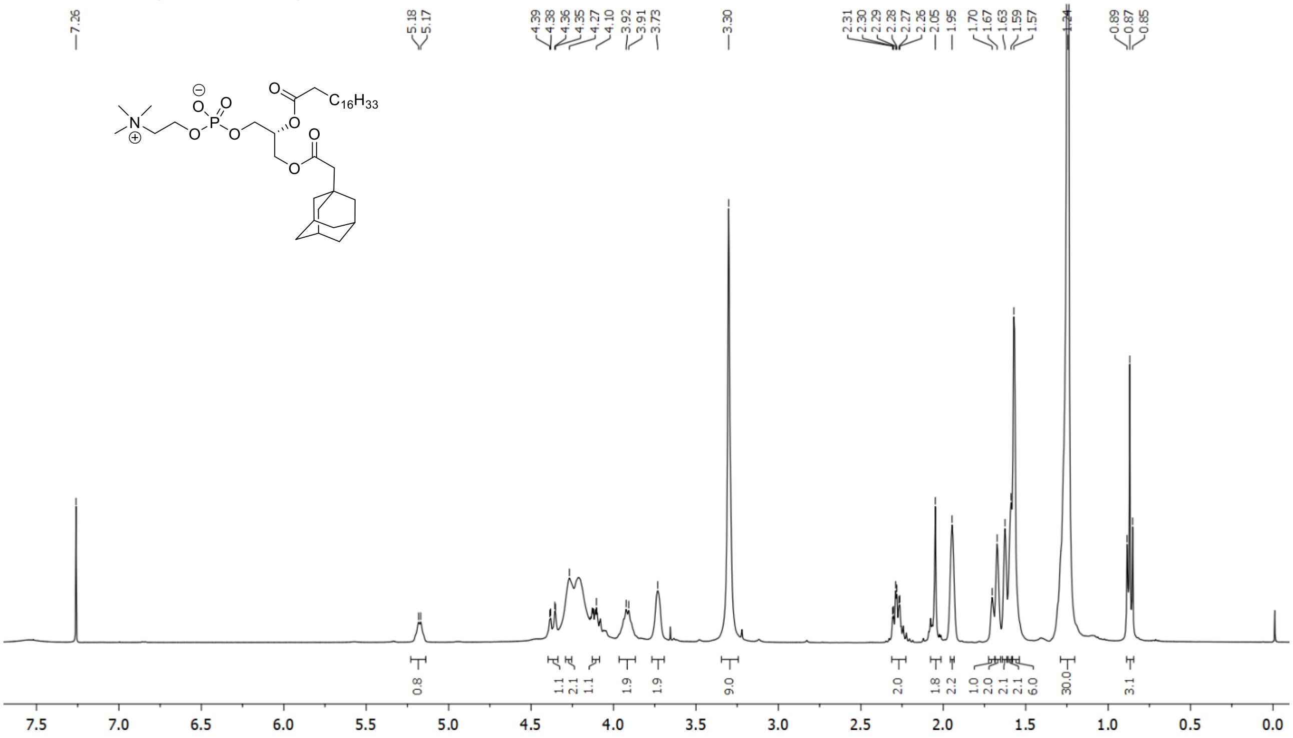

A-17

^{13}C-NMR (100 MHz, $CDCl_3$)

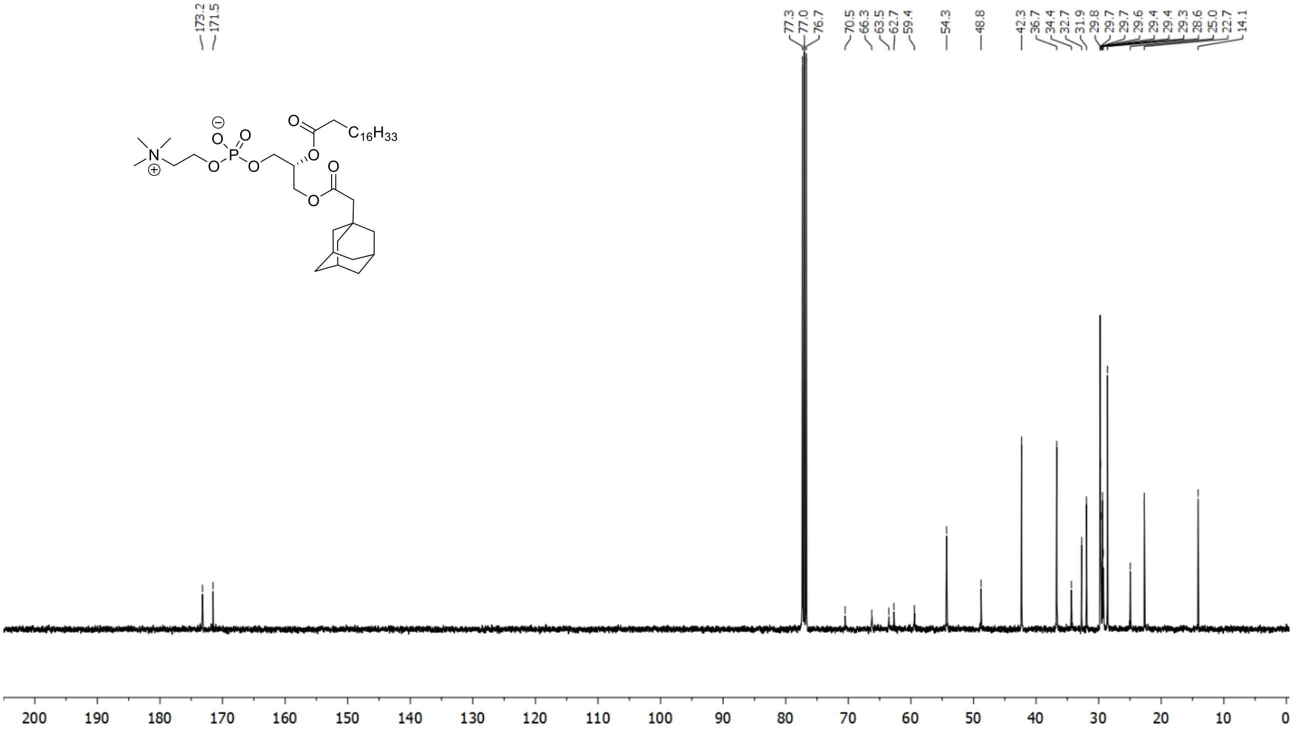

A-17-2*Z*
^{1}H-NMR (400 MHz, $CDCl_3$)

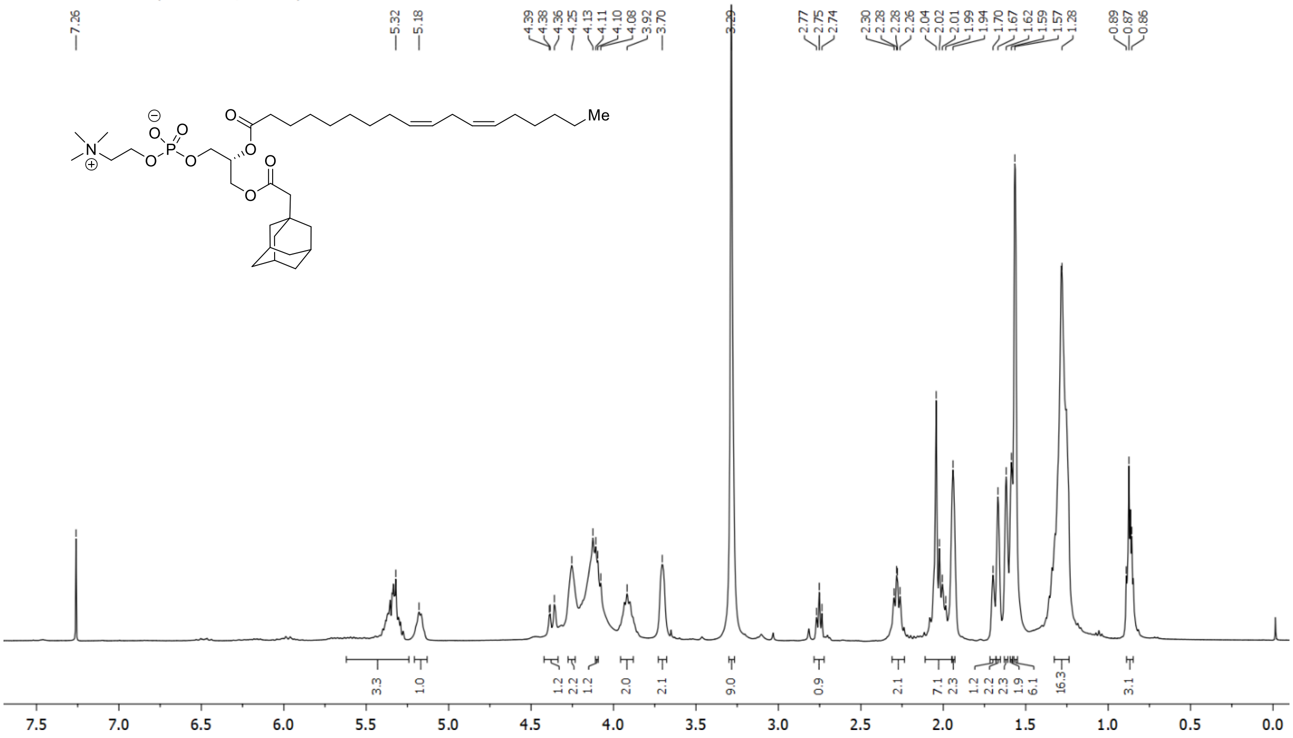

A-17-2*Z*
^{13}C-NMR (100 MHz, $CDCl_3$)

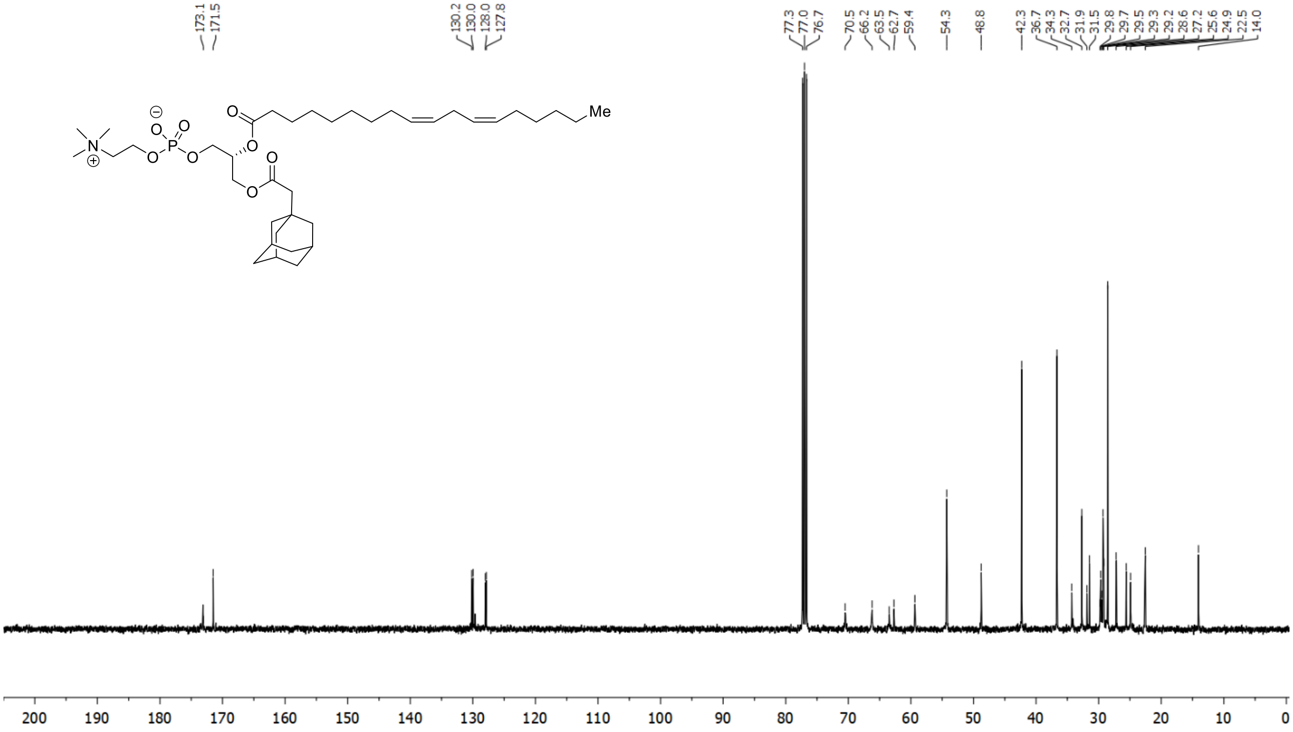

jd190212-02 #604-624 RT: 4.77-4.93 AV: 21 NL: 3.78E7
T: FTMS + p ESI Full ms [100.00-2000.00]

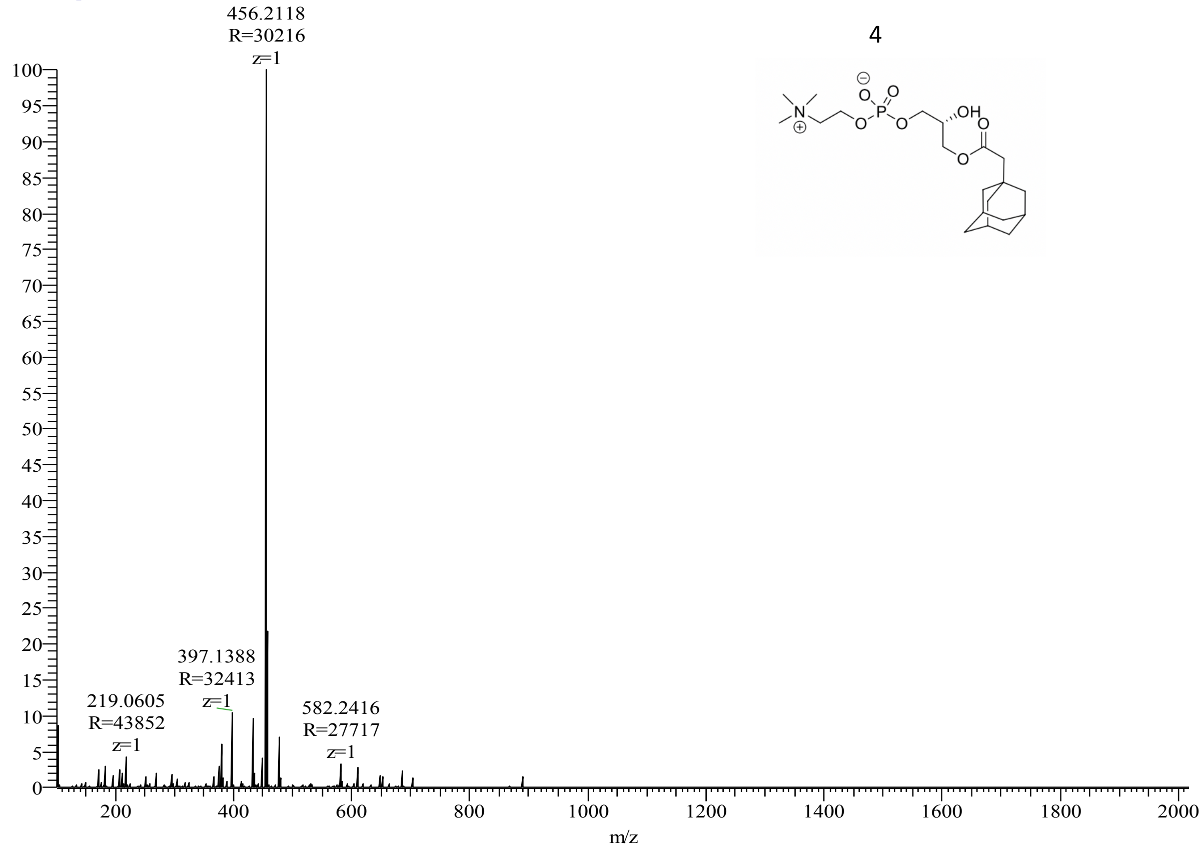

4

jd190222-01 #190-213 RT: 1.50-1.69 AV: 24 NL: 1.14E7
T: FTMS + p ESI Full ms [150.00-2000.00]

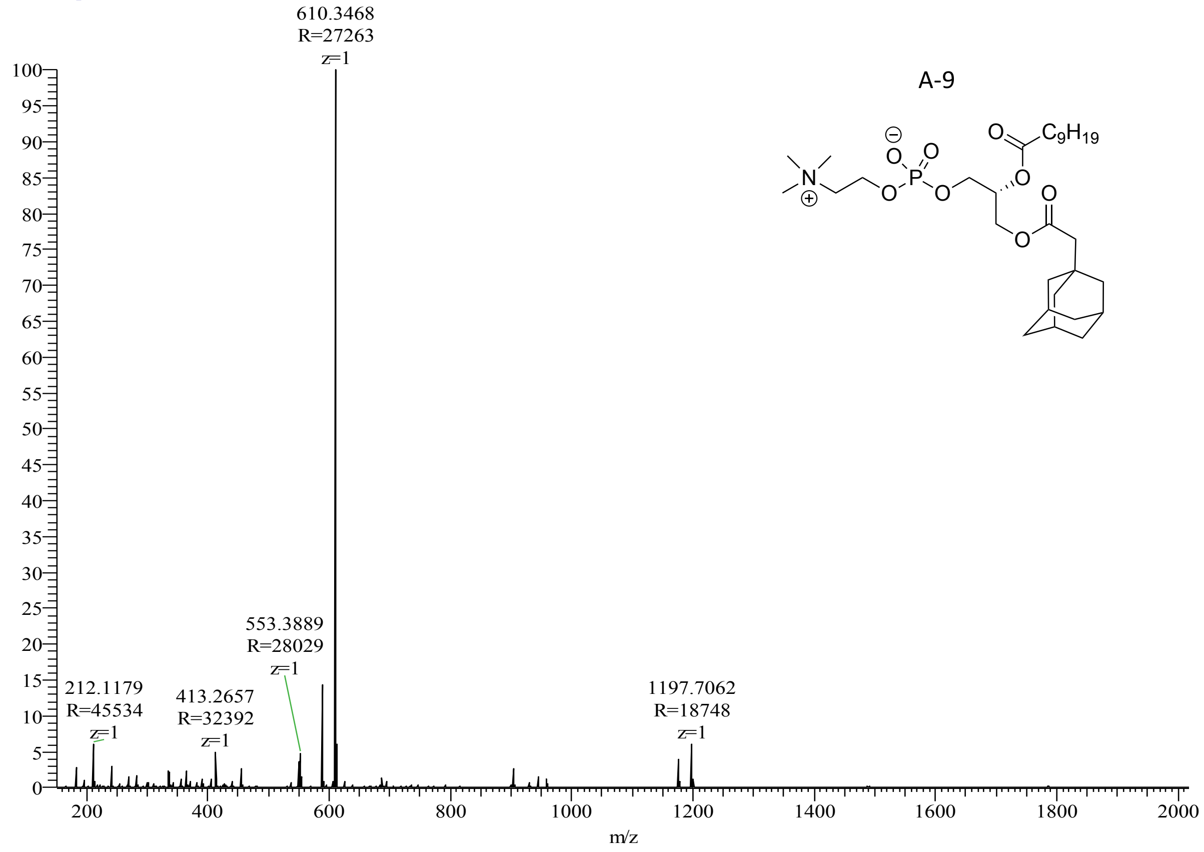

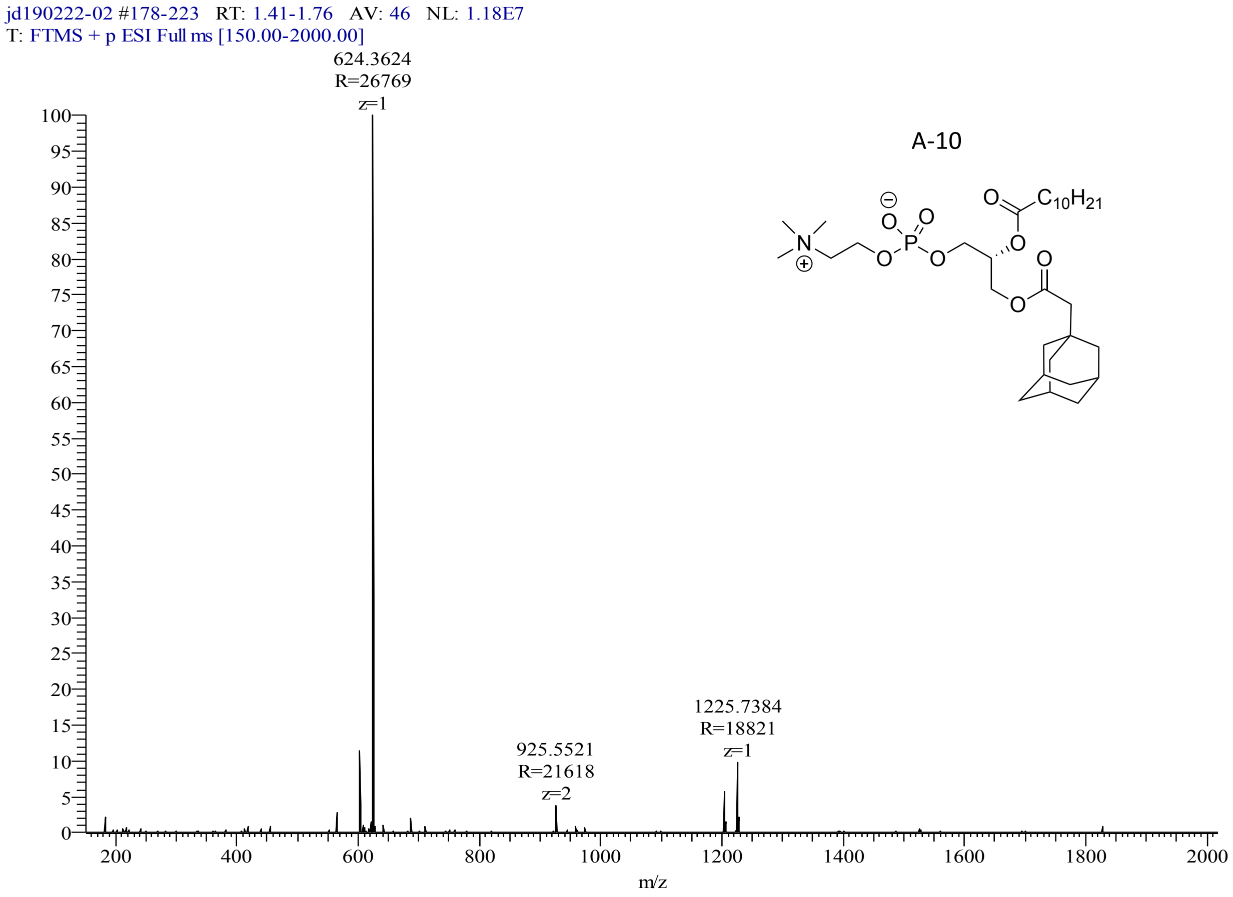
jd190222-02 #178-223 RT: 1.41-1.76 AV: 46 NL: 1.18E7
T: FTMS + p ESI Full ms [150.00-2000.00]
624.3624
R=26769
z=1
925.5521
R=21618
z=2
1225.7384
R=18821
z=1
m/z
A-10
C10H21

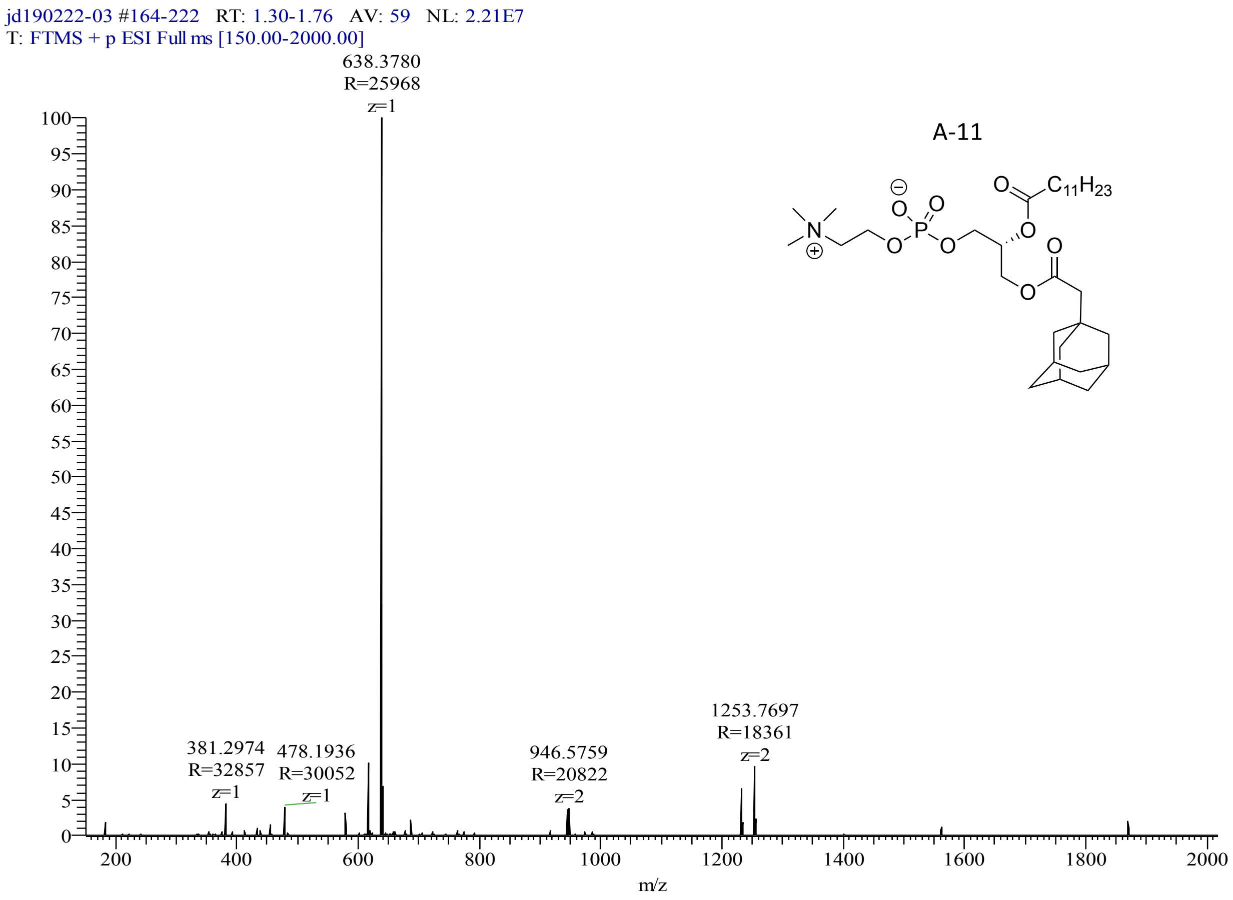
jd190222-03 #164-222 RT: 1.30-1.76 AV: 59 NL: 2.21E7
T: FTMS + p ESI Full ms [150.00-2000.00]
638.3780
R=25968
z=1
381.2974
R=32857
z=1
478.1936
R=30052
z=1
946.5759
R=20822
z=2
1253.7697
R=18361
z=2
m/z
A-11

jd190222-04 #172-216 RT: 1.36-1.71 AV: 45 NL: 2.82E6
T: FTMS + p ESI Full ms [150.00-2000.00]

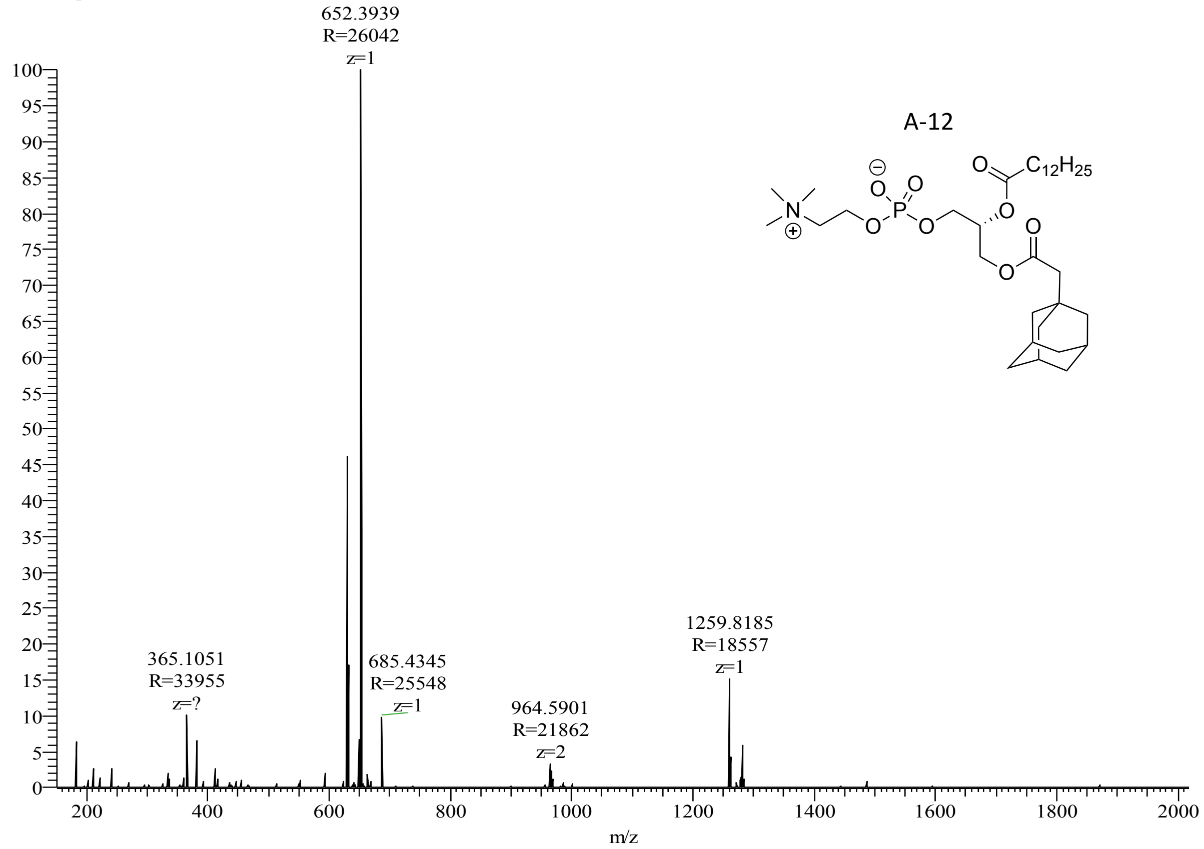

A-13

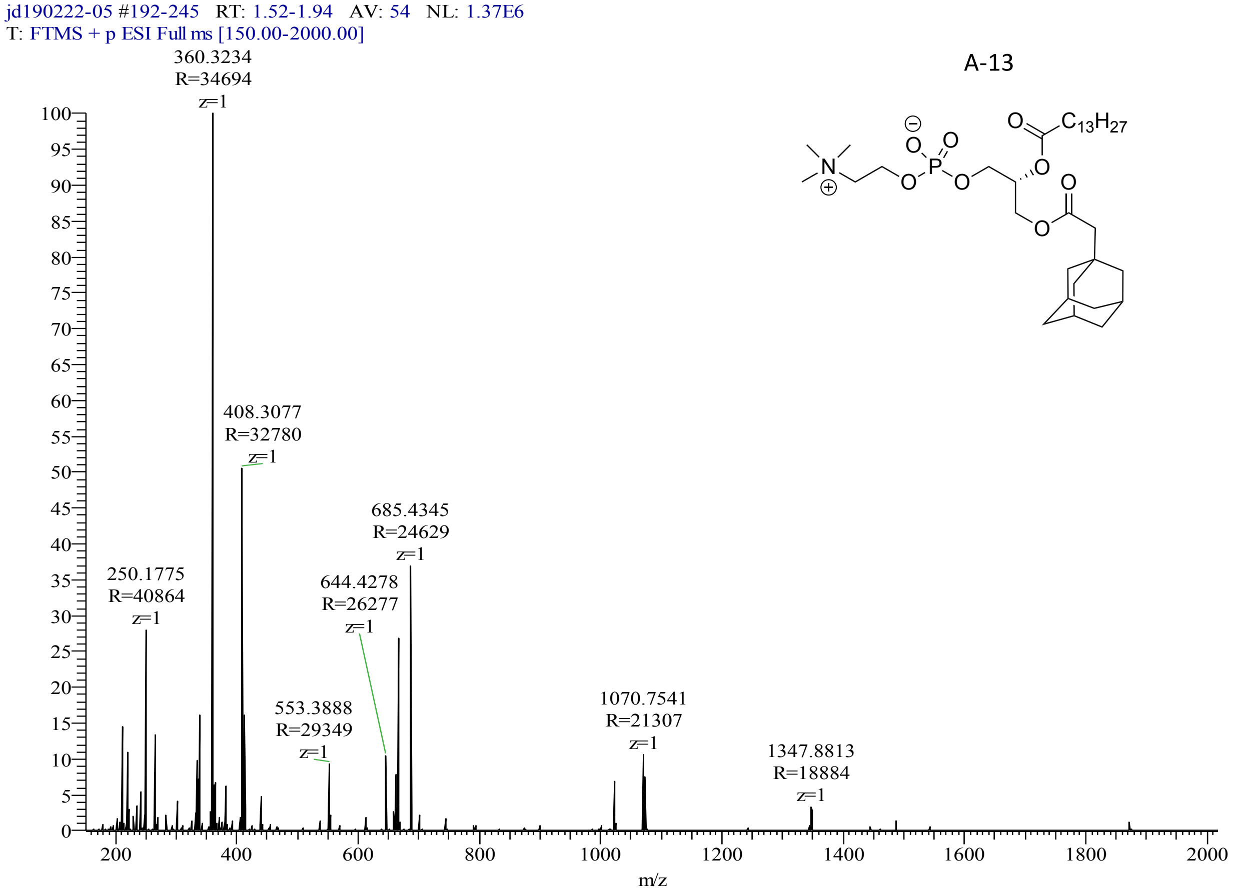
jd190222-05 #192-245 RT: 1.52-1.94 AV: 54 NL: 1.37E6
T: FTMS + p ESI Full ms [150.00-2000.00]
360.3234
R=34694
z=1
408.3077
R=32780
z=1
685.4345
R=24629
z=1
250.1775
R=40864
z=1
644.4278
R=26277
z=1
553.3888
R=29349
z=1
1070.7541
R=21307
z=1
1347.8813
R=18884
z=1
m/z
O
C13H27
O
O
P
O
O
N
O
O
O

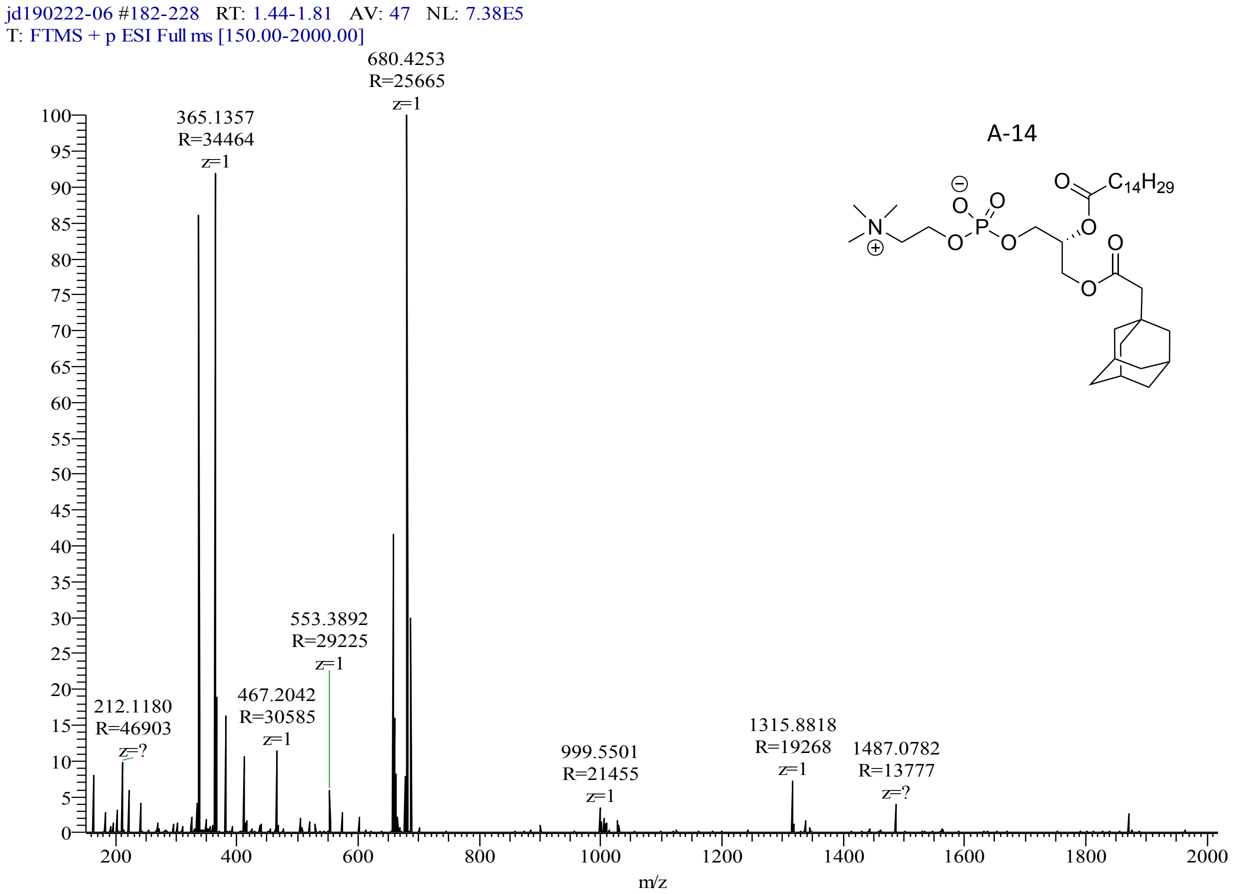
jd190222-06 #182-228 RT: 1.44-1.81 AV: 47 NL: 7.38E5
T: FTMS + p ESI Full ms [150.00-2000.00]
680.4253
R=25665
z=1
365.1357
R=34464
z=1
553.3892
R=29225
z=1
212.1180
R=46903
z=?
467.2042
R=30585
z=1
999.5501
R=21455
z=1
1315.8818
R=19268
z=1
1487.0782
R=13777
z=?
m/z

A-14

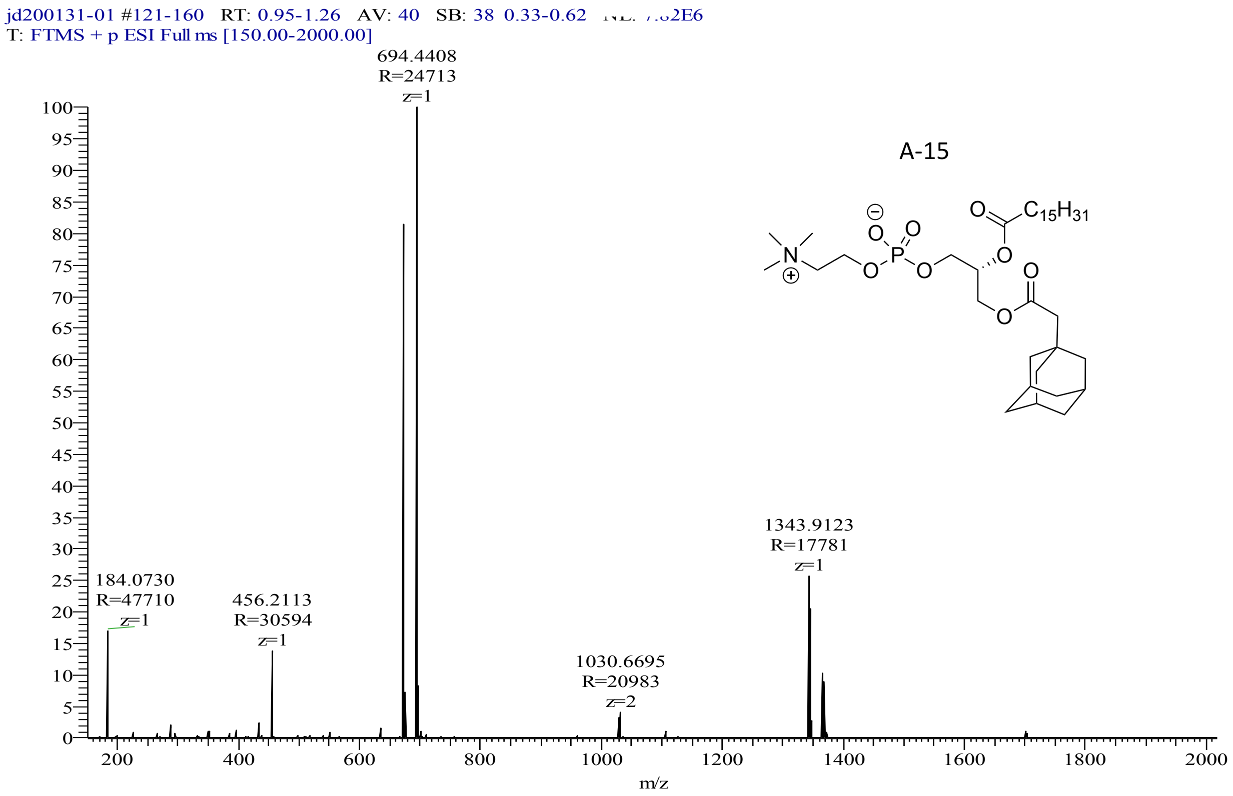
jd200131-01 #121-160 RT: 0.95-1.26 AV: 40 SB: 38 0.33-0.62
T: FTMS + p ESI Full ms [150.00-2000.00]
694.4408
R=24713
z=1
A-15
$C_{15}H_{31}$
1343.9123
R=17781
z=1
184.0730
R=47710
z=1
456.2113
R=30594
z=1
1030.6695
R=20983
z=2
m/z

jd190222-07 #182-220 RT: 1.44-1.75 AV: 39 NL: 4.87E5
T: FTMS + p ESI Full ms [150.00-2000.00]

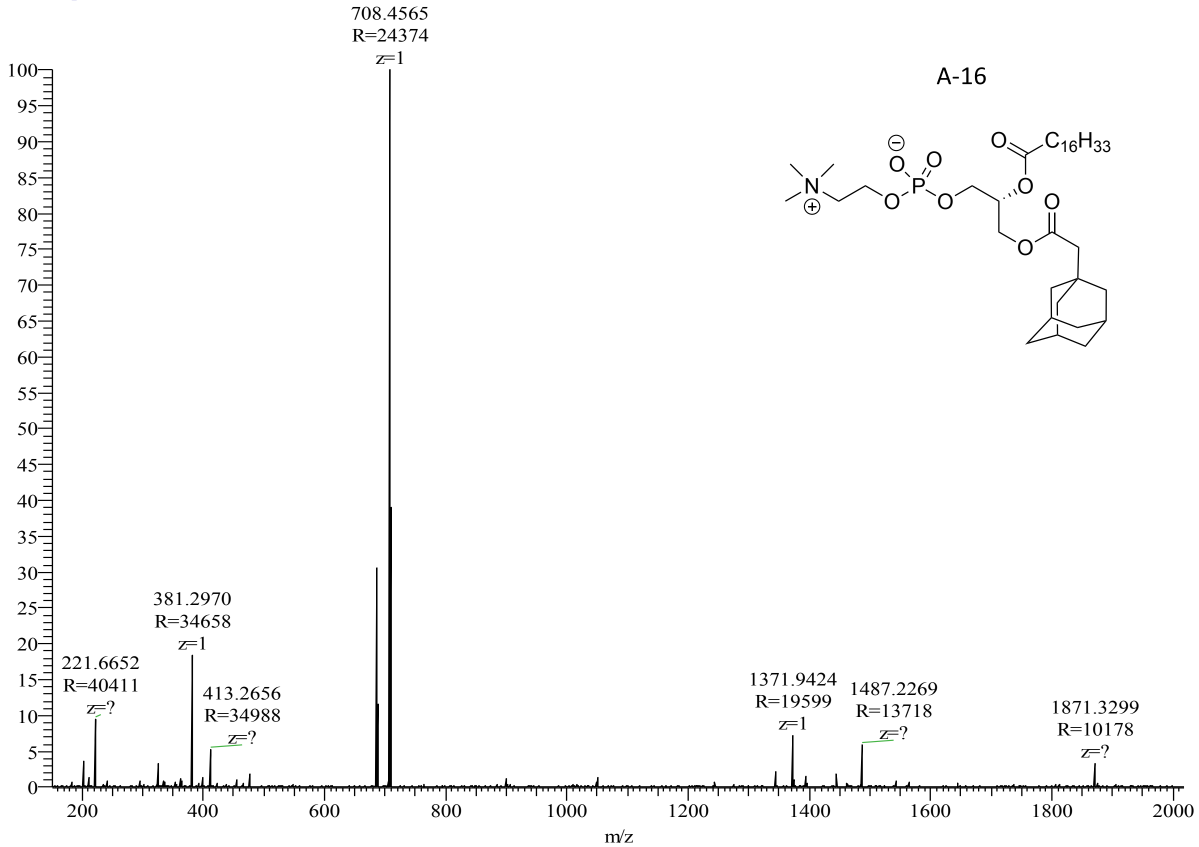

jd190222-08 #173-215 RT: 1.37-1.70 AV: 43 NL: 1.30E6
T: FTMS + p ESI Full ms [150.00-2000.00]

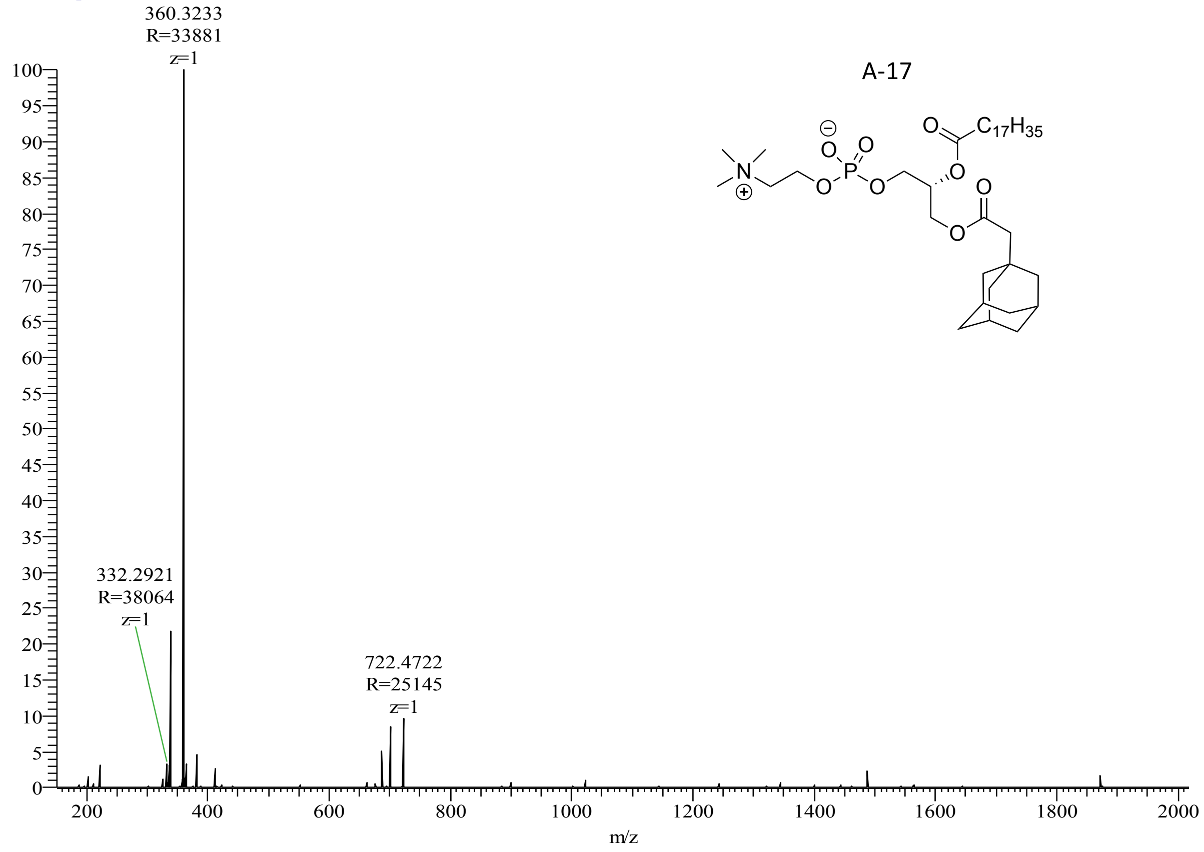

A-17

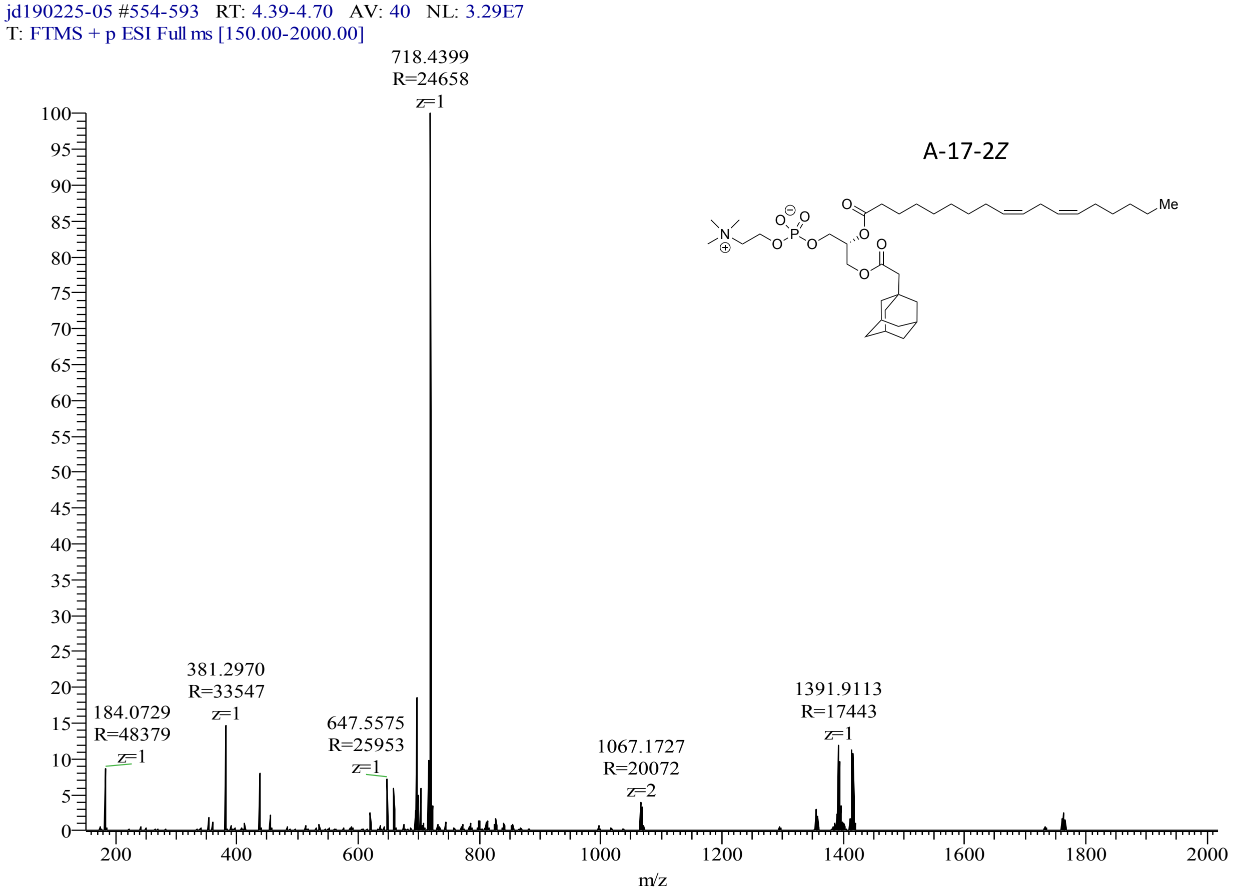
jd190225-05 #554-593 RT: 4.39-4.70 AV: 40 NL: 3.29E7
T: FTMS + p ESI Full ms [150.00-2000.00]
718.4399
R=24658
z=1
A-17-2Z
Me
381.2970
R=33547
z=1
184.0729
R=48379
z=1
647.5575
R=25953
z=1
1067.1727
R=20072
z=2
1391.9113
R=17443
z=1
m/z

References:

1. Garcia-Garibay, M. et. al. Mechanistic Studies of Adamantylacetophenones with Competing Reaction Pathways in Solution and in the Crystalline Solid State. The Journal of Organic Chemistry 84 (17), 11103-11113 (2019)
2. Dahlman JE, et al. Barcoded nanoparticles for high throughput in vivo discovery of targeted therapeutics. Proc Natl Acad Sci USA 114:2060–2065. (2017)
3. Paunovska K, et al. A direct comparison of in vitro and in vivo nucleic acid delivery mediated by hundreds of nanoparticles reveals a weak correlation. Nano Lett 18:2148–2157 (2018)
4. Dahlman, James E. et. al. In vivo endothelial siRNA delivery using polymeric nanoparticles with low molecular weight. Nat Nanotechnol. 2014 Aug; 9(8): 648–655.
5. Sager HB, et al. (2016) RNAi targeting multiple cell adhesion molecules reduces immune cell recruitment and vascular inflammation after myocardial infarction. Science Transl Med 8:342ra380.
